# Divergent Association Pathways of Soil Organic Carbon Variation Under Grazing Exclusion Across Three Grassland Sites: Relationships with Microbial Network Structure and Community Assembly

**DOI:** 10.3390/microorganisms14092023

**Published:** 2026-09-11

**Authors:** Asitaiken Julihaiti, Zongjiu Sun, Jinhua Guo, Yisheng Jing, Yiqiang Dong

**Affiliations:** 1 School of Grassland, Xinjiang Agricultural University, Urumqi 830052, China; astekin77@163.com (A.J.);; 2 Key Laboratory of Grassland Resources and Ecology of Xinjiang Uygur Autonomous Region, Urumqi 830052, China; 3 Key Laboratory of Grassland Resources and Ecology of Western Arid Region, Ministry of Education, Urumqi 830052, China

**Keywords:** grazing exclusion, microbial network complexity, microbial community assembly processes, soil organic carbon

## Abstract

Grazing exclusion is a widely implemented restoration strategy for degraded grasslands, but its effects on soil organic carbon (SOC) remain highly variable, and the ecological pathways associated with these changes—particularly the roles of soil microorganisms—are still insufficiently understood. To investigate context-dependent SOC responses, we conducted a 12-year grazing exclusion study across three contrasting grassland sites representing an environmental gradient (temperate desert, temperate steppe, and mountain meadow) on the northern slope of the Tianshan Mountains. By integrating multidimensional vegetation–soil–microbe observations with microbial network analysis and community assembly approaches, we examined how plant, soil, and microbial characteristics were associated with SOC variation under grazing exclusion. Our results showed that grazing exclusion was associated with significant increases in surface SOC content across all three investigated sites (16–81%), but these increases corresponded to distinct site-specific ecological association patterns. At the temperate desert site, SOC variation was mainly associated with soil conditions and microbial characteristics, where soil moisture and nutrient availability, together with fungal network complexity, showed significant associations with SOC content. At the temperate steppe site, vegetation recovery represented an important ecological component associated with SOC variation, while bacterial community assembly patterns were also related to SOC differences. At the mountain meadow site, plant, soil, and microbial characteristics showed multiple associations with SOC variation. Across all sites, grazing exclusion was consistently associated with changes in microbial network structure and community assembly processes, and these microbial ecological characteristics showed significant statistical relationships with SOC content. Our findings indicate that SOC responses to grazing exclusion are context-dependent and involve different ecological association pathways across contrasting grassland ecosystems, highlighting the importance of considering local environmental conditions when evaluating grassland restoration outcomes and carbon management strategies.

## 1. Introduction

Soils store the largest terrestrial organic carbon pool, holding approximately three times more carbon than the vegetation pool [1]. Grasslands, covering vast areas globally, account for about 30–34% of global soil organic carbon (SOC), playing a vital role in climate regulation and biodiversity maintenance [2,3]. However, nearly half of the world’s grasslands have suffered degradation due to overgrazing, severely compromising their ecosystem services and carbon sink potential [4]. Grazing exclusion, a cornerstone restoration measure [5], effectively promotes vegetation recovery [6]. Yet, its impact on the soil organic carbon content remains highly contentious, with reported outcomes ranging from significant increases to neutral or even negative effects [7,8]. This uncertainty strongly suggests that the effect on surface SOC content of grazing exclusion is not universal but governed by context-dependent mechanisms [9].

Resolving this macro-scale controversy requires a better understanding of the micro-scale ecological processes associated with SOC dynamics. Since SOC dynamics are closely linked to microbial-mediated processes [10,11], the effects of grazing exclusion are expected to be reflected in changes in soil microbial community structure and ecological functions. Previous research has predominantly focused on microbial composition and diversity [12,13] or simple correlations with environmental factors. However, less attention has been given to two important ecological pathways that may help explain associations between microbial ecological characteristics and SOC variation: (1) the structure of microbial co-occurrence networks formed through interspecific interactions, and (2) the ecological processes governing community assembly, including the relative roles of deterministic and stochastic processes.

Specifically, complex microbial co-occurrence networks, which describe correlation-based associations among microbial taxa, have been suggested to reflect aspects of microbial community organization and may be associated with ecosystem functioning [14]. Although previous studies have demonstrated that grazing exclusion can alter microbial networks [15,16], the direction and magnitude of these changes—whether increasing or decreasing network complexity—remain inconsistent among ecosystems [17,18], suggesting that microbial network responses may depend strongly on environmental context. Moreover, previous research has often focused on single sites or individual microbial kingdoms, with relatively limited comparisons of bacterial and fungal networks, despite their potentially distinct ecological strategies and responses to grazing exclusion [19]. Meanwhile, microbial community assembly processes determine microbial community structure and ecological functions, reflecting ecological processes that differ from those represented by network characteristics [20]. These assembly processes, shaped by both deterministic (e.g., environmental filtering) and stochastic (e.g., dispersal and ecological drift) forces, may be altered by anthropogenic disturbances [21]. However, comparative analyses of bacterial and fungal assembly responses under grazing exclusion across contrasting grassland sites remain limited, leaving uncertainty regarding whether these microbial responses are consistent or decoupled [22,23].

Notably, microbial network characteristics and community assembly processes represent complementary aspects of microbial ecological states and may jointly be associated with ecosystem functions [24]. However, under grazing exclusion, how changes in microbial assembly processes are associated with network restructuring and further relate to SOC variation—the relationship between microbial ecological states and SOC variation—remains insufficiently understood.

The above analysis highlights an important unresolved question: do grazing exclusion effects on microbial network properties and community assembly processes vary among contrasting grassland sites with different environmental backgrounds, and are these variations associated with divergent SOC-related pathways? The three investigated sites in this study represent a precipitation and resource availability gradient, ranging from extremely arid conditions (TD) to semi-arid conditions (TS) and relatively humid conditions (MM), with distinct initial soil, vegetation, and climatic characteristics. Therefore, comparing these sites provides an opportunity to evaluate how grazing exclusion is associated with microbial ecological changes and SOC variation under different environmental contexts.

Building upon this framework, we hypothesized that the associations between grazing exclusion and SOC variation would differ among the three investigated sites due to contrasting environmental constraints and ecosystem conditions. Specifically, we expected that SOC-associated pathways would vary along the environmental gradient, with stronger associations with soil conditions at the arid site, stronger associations with vegetation recovery at the semi-arid site, and multiple plant–soil–microbe associations under relatively favorable environmental conditions at the humid site.

Accordingly, this study aimed to: (1) determine how grazing exclusion is associated with bacterial and fungal co-occurrence network characteristics among three contrasting grassland sites; (2) characterize differences in bacterial and fungal community assembly processes among these sites; and (3) evaluate the relative associations of plant, soil, and microbial characteristics with SOC variation under grazing exclusion. By integrating microbial co-occurrence networks and community assembly processes, we aim to provide a microbial ecological perspective for understanding how grazing exclusion is associated with microbial ecological changes and SOC variation under different environmental contexts.

## 2. Materials and Methods

### 2.1. Study Area and Experimental Design

**Figure 1:**
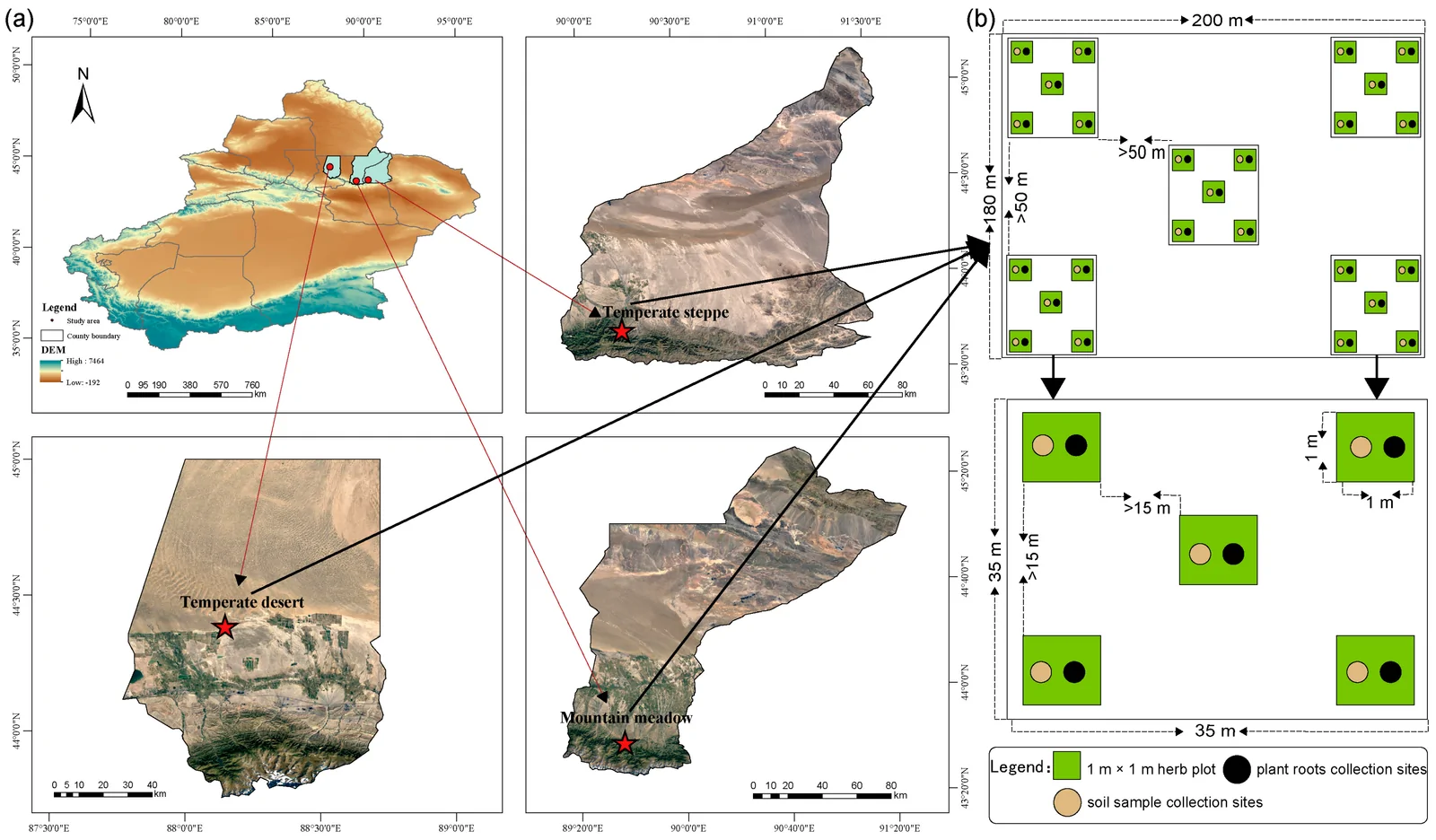
Location of the study area and experimental design of the grazing exclusion plots. Note: (**a**) Geographic location of the sampling sites across the three grassland types in Xinjiang, China. (**b**) Schematic illustration of the five-point sampling method used within each quadrat.

The study area is situated on the middle section of the northern slope of the Tianshan Mountains in Xinjiang, covering Fukang City, Mulei County, and Qitai County within the Changji Hui Autonomous Prefecture, where the basic characteristics of sampling sites are summarized in Figure 1. Specifically, the Fukang area (88°08′ E, 44°23′ N; 514.0 m a.s.l.; mean annual precipitation 145.0 mm) is characterized by a temperate desert with sierozem soil (classified as Calcisol according to the World Reference Base for Soil Resources, WRB) of sandy texture, dominated by *Haloxylon ammodendron* and *Seriphidium santolinum* and associated with *Salsola collina*. In contrast, the Mulei area (90°14′ E, 43°40′ N; 1987 m a.s.l.; mean annual precipitation 296.6 mm) features a temperate steppe with chernozem (Chernozem) or chestnut soil (Kastanozem) (both WRB) of loam texture, mainly dominated by *Stipa capillata*, *Festuca ovina*, and *Carex liparocarpos* and associated with *Sibbaldianthe bifurca*. Furthermore, the Qitai area (889°35′ E, 43°37′ N; 2093 m a.s.l.; mean annual precipitation 420 mm) is identified as a mountain meadow with chernozem soil (Chernozem according to WRB) of loam texture, primarily dominated by *Achillea millefolium*, *Geranium carolinianum*, and *Thalictrum aquilegiifolium* and associated with species such as *Codonopsis pilosula*, *Leontopodium leontopodioides*, and *Campanula glomerata*.

The test sites were strategically positioned within the designated national fixed monitoring sites, each encompassing an area of approximately 3 ha. Two treatments are set in temperate desert (TD), temperate steppe (TS), and mountain meadow (MM), respectively: grazing exclusion plots (GE) and freely grazing plots (FG). Notably, all these exclusion plots have been enclosed since 2012 and remained enclosed for a duration of 12 years by the sampling year of 2024. The overall stocking rate for the three grassland sites ranged from 0.90 to 4.50 sheep units per hectare. The grazing intensities at these three sites fell within the category of moderate grazing in the local context, aligning with the carrying capacity of livestock in the area. Before the implementation of grazing exclusion, the areas designated for grazing exclusion (GE) exhibited comparable vegetation composition, community characteristics, and landforms to the free-grazing (FG) plots.

### 2.2. Plant and Soil Sampling and Determination

Field investigations were conducted in late June 2024. At each grassland site, a long-term grazing-exclusion area and an adjacent freely grazed area were selected within the same fixed monitoring station. The grazing-exclusion and freely grazed areas were located within several tens of meters of each other to minimize differences in topography and background environmental conditions. Within each treatment area, five spatially separated plots (35 m × 35 m each) were established with an interval of more than 50 m (Figure 1). Within each plot, five 1 m × 1 m quadrats were arranged according to the five-point method, with a distance of approximately 15 m between quadrats, resulting in a total of 150 quadrats sampled. In each quadrat, plant species were recorded, and coverage (%), height (cm), density (plants·m^−2^), and biomass were measured. Above-ground biomass (AGB) was harvested, oven-dried (105 °C for 30 min, then 80 °C for 24 h), and weighed. Below-ground biomass (BGB) was determined from intact soil blocks (10 × 20 cm) after washing and drying, following the methods of [25].

For soil sampling, a soil auger was used to collect samples from the 0–10 cm layer within each vegetation quadrat. The five subsamples collected from the five quadrats within the same plot were thoroughly mixed to form one composite sample per plot. Therefore, each 35 m × 35 m plot represented one sampling unit for subsequent analyses. Each composite sample was sealed in a labeled bag and transported to the laboratory. One subset was stored at −20 °C for microbial analysis; the remainder was air-dried, sieved (2 mm, 1 mm, 0.25 mm), and stored for physicochemical analysis. Thus, five spatially separated sampling plots were used to characterize spatial variation within each treatment area.

Soil pH (soil:water = 5:1) and soil water content (SWC) were measured electrometrically and by gravimetry, respectively. Soil organic carbon (SOC), total nitrogen (TN), and total phosphorus (TP) were determined using potassium dichromate oxidation-heating, Kjeldahl digestion, and molybdenum-antimony colorimetry methods, respectively [26]. Microbial biomass carbon (MBC), nitrogen (MBN), and phosphorus (MBP) were analyzed using the chloroform fumigation–extraction method [27,28].

### 2.3. Soil Microbial Sampling and Determination

Within the surveyed grassland community quadrats, soil microbial samples were collected from the 0–10 cm soil layer (Figure 1). Samples from each transect were thoroughly homogenized, placed in sealed bags, and stored at −20 °C for transport to the laboratory.

Total microbial DNA was extracted using a commercial metagenomic DNA extraction kit. Bacterial communities were characterized by amplifying the V3–V4 region of the bacterial 16S rRNA gene using primers 338F/806R, while fungal communities were characterized by amplifying the ITS1 region using ITS1F/ITS1R primers. PCR products were purified, quantified, and used for library construction. Sequencing was performed on the Illumina MiSeq PE300 platform by Nanjing Jice Biotechnology Co., Ltd. (Nanjing, China).

Raw sequencing reads were processed using the QIIME2 pipeline. Quality filtering was performed by removing low-quality reads, sequences containing ambiguous bases, and short reads. Chimeric sequences were removed during sequence processing. Amplicon sequence data were clustered into operational taxonomic units (OTUs) at a 97% similarity threshold, and representative sequences were taxonomically assigned using the SILVA database for bacteria and the UNITE database for fungi. OTUs with extremely low abundance (<0.001% of total sequences) were removed before downstream analyses. Microbial diversity indices, including richness and Shannon diversity, were calculated based on the processed OTU table.

Phylogenetic trees used for microbial community assembly analyses were generated from representative sequences using the QIIME2 pipeline. The raw sequence data have been deposited in the NCBI BioProject database (accession number: PRJNA1445889).

### 2.4. Construction of Soil Microbial Co-Occurrence Network

To assess changes in microbial co-occurrence patterns associated with grazing exclusion, microbial co-occurrence networks were constructed separately for bacterial and fungal communities under grazing exclusion and free grazing conditions across the three investigated sites (i.e., Fukang, Mulei, and Qitai). For each combination of site, treatment, and microbial group, one reference network was constructed, resulting in 12 reference networks in total. After filtering OTUs with relative abundances below 0.001%, co-occurrence networks were constructed at the OTU level based on OTU relative abundance profiles of bacterial and fungal taxa. Pairwise Spearman correlations among OTUs were calculated using the “psych” package in R, and correlation matrices were transformed into edge lists using the “reshape2” package. Significant correlations (|R| > 0.7, FDR-adjusted *p* < 0.05) were retained as network edges, and network visualization was performed using Gephi 0.10.1.

Network topological properties were subsequently calculated using the “igraph” package in R. To evaluate treatment effects, individual sample-level subnetworks were extracted from the corresponding reference networks using the “induced_subgraph” function based on OTU occurrence patterns in each sample. The topological features of each subnetwork were standardized using Z-score normalization, and network complexity was quantified as the mean value of five normalized topological parameters, including the number of nodes, number of edges, average degree, average clustering coefficient, and modularity [29]. Therefore, each treatment within each investigated site generated five sample-level network-property values (n = 5), which were subsequently used for statistical comparisons between grazing exclusion and free grazing treatments. Furthermore, based on within-module connectivity (Zi) and among-module connectivity (Pi), network nodes were classified into four topological categories: peripherals (Zi < 2.5, Pi < 0.62), connectors (Zi < 2.5, Pi > 0.62), network hubs (Zi > 2.5, Pi > 0.62), and module hubs (Zi > 2.5, Pi < 0.62). Nodes with high Zi or Pi values were identified as potential keystone taxa [30,31].

### 2.5. Analysis of Soil Microbial Community Assembly Processes

The assembly mechanisms of bacterial and fungal communities were assessed using phylogenetic null model analysis. This approach was primarily implemented based on the analytical framework proposed by Zhou and Ning [20], with reference to the methodologies established by Stegen et al. [32,33] and Dini-Andreote et al. [34].

Phylogenetic trees of bacterial and fungal communities were generated based on representative sequences using the QIIME2 pipeline. The phylogenetic null model analysis was conducted using the “picante” package in R to quantify phylogenetic turnover among microbial communities. Specifically, abundance-weighted β-mean nearest taxon distance (βMNTD) was calculated, and β-nearest taxon index (βNTI) values were obtained by comparing observed βMNTD values with null distributions generated from 1000 randomizations. The Bray–Curtis-based Raup–Crick index (RCBray) was further calculated using 500 randomizations to distinguish stochastic assembly processes.

The βNTI represents the standardized deviation of observed phylogenetic turnover from the null expectation. Following the framework of Stegen et al. [32,33], deterministic processes were inferred when |βNTI| > 2, including homogeneous selection (βNTI < −2) and heterogeneous selection (βNTI > +2). When |βNTI| < 2, stochastic processes were further classified using RCBray values: RCBray < −0.95 indicated homogenizing dispersal, RCBray > +0.95 indicated dispersal limitation, and |RCBray| < 0.95 represented undominated processes (e.g., weak selection or weak dispersal) [35,36].

Because βNTI and RCBray are pairwise metrics calculated between microbial communities, ecological assembly processes were assigned based on all pairwise comparisons among samples. The relative contribution of each assembly process was calculated as the proportion of pairwise comparisons classified into each ecological process category. Therefore, these results represent overall community assembly patterns at the treatment level rather than independent sample-level measurements.

Because fungal ITS sequences generally have lower phylogenetic resolution than bacterial 16S rRNA gene sequences, fungal βNTI results were interpreted cautiously as indicators of relative phylogenetic turnover rather than direct evidence of evolutionary constraints.

### 2.6. Data Statistics and Analysis

Primary data preprocessing was performed using Microsoft Excel 2020 (Microsoft Corp., Redmond, WA, USA). To evaluate differences in plant traits, soil characteristics, microbial properties, and microbial network topological parameters between grazing exclusion (GE) and freely grazed (FG) treatments, statistical analyses were conducted using IBM SPSS Statistics 25.0 (IBM Corp., Armonk, NY, USA). A hierarchical analytical framework was applied: (1) Before parametric tests, data normality was assessed using the Shapiro–Wilk test, and homogeneity of variances was evaluated using Levene’s test (α = 0.05). When data met normality assumptions and variances were homogeneous, Student’s *t*-test was applied, whereas Welch’s *t*-test was used when variances were unequal. For variables violating normality assumptions, Mann–Whitney U tests were performed; (2) Differences between GE and FG treatments within each investigated site were evaluated using appropriate two-sided statistical tests based on the above assumptions, with five spatial sampling plots per treatment. Given that each grassland type was represented by a single geographical site, these comparisons were interpreted as site-specific treatment associations rather than universal effects across all grasslands of the same type. For comparisons among grassland types within the same treatment, one-way analysis of variance (ANOVA) followed by Tukey’s HSD test was conducted when assumptions of normality and homogeneity of variance were satisfied.

Subsequently, statistical analyses in R included: (1) Spearman correlation analyses were performed to examine associations among microbial co-occurrence network properties, community assembly processes, and environmental variables, and *p* values were adjusted using the Benjamini–Hochberg false discovery rate (FDR) correction; and (2) Random forest modeling was conducted as an exploratory analysis to evaluate the relative importance of plant traits, soil physicochemical properties, and microbial characteristics associated with SOC variation. Given the limited sample size, random forest results were interpreted as exploratory estimates of variable importance rather than predictive performance.

Furthermore, Partial Least Squares Structural Equation Modeling (PLS-SEM) was performed using the plspm package (v0.4.9) in R 4.2.1 as an exploratory framework to evaluate potential direct and indirect associations among grazing exclusion, plant characteristics, soil properties, microbial properties, and SOC variation. The models incorporated key soil properties (pH, SWC, TN, and TP), plant characteristics (AGB, BGB, richness, and Shannon–Wiener index), and microbial properties (MBC, MBN, and MBP). Separate models were constructed for each investigated site to explore site-specific association patterns related to SOC variation under grazing exclusion. Given the limited number of spatial replicates, PLS-SEM results were interpreted cautiously and were not considered evidence of causal relationships. The coefficient of determination (R^2^) and Goodness-of-Fit (GoF) index were calculated as descriptive indicators of model structure rather than independent validation criteria. The uncertainty of path coefficients was estimated through bootstrap resampling with 5000 iterations.

Finally, all statistical graphics were generated using ggplot2 (v3.4.2) in R 4.2.1. Subsequent vector refinement and compositional optimization were performed using Adobe Illustrator 2021 (Adobe Inc., San Jose, CA, USA). Data are presented as mean ± standard error (SE).

## 3. Results

### 3.1. Effects of Grazing Exclusion on Soil Microbial Co-Occurrence Networks and Complexity

**Figure 2:**
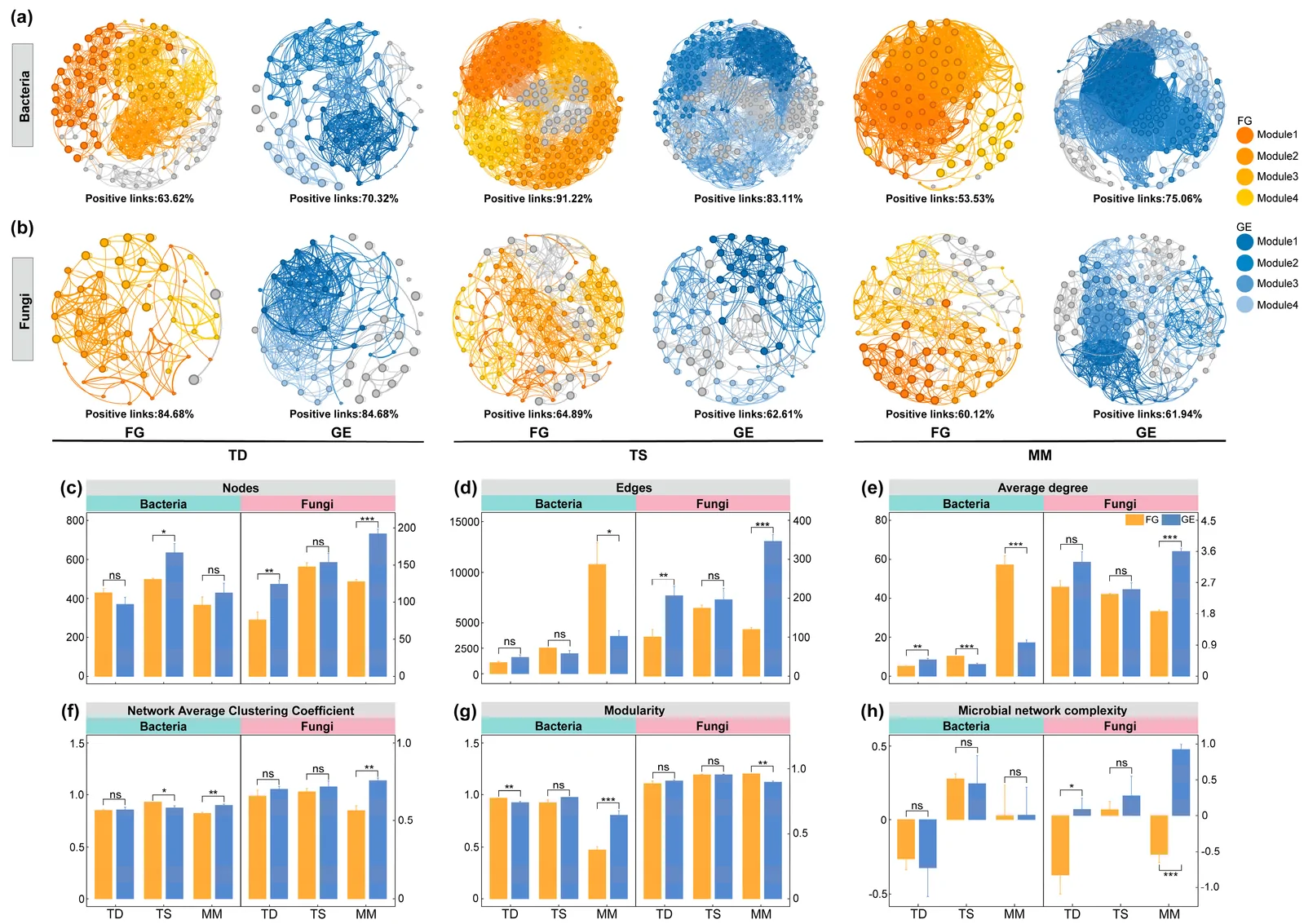
Changes in soil microbial co-occurrence networks and their complexity in response to grazing exclusion. (**a**) Bacterial co-occurrence networks under freely grazed (FG) and grazing exclusion (GE) treatments across the three grassland types. (**b**) Fungal co-occurrence networks under FG and GE treatments. (**c**) Number of nodes. (**d**) Number of edges. (**e**) Average degree. (**f**) Network average clustering coefficient. (**g**) Modularity. (**h**) Microbial network complexity.Note: All values and error bars represent mean ± standard error of network topological parameters calculated from sample-level subnetworks (n = 5 per treatment). Significant differences between grazing exclusion and freely grazed treatments within each grassland type were determined using independent-sample *t* tests. “*”, “**”, and “***” indicate significance levels of *p* < 0.05, *p* < 0.01, and *p* < 0.001, respectively, whereas “ns” indicates no significant difference (*p* > 0.05). TD, temperate desert; TS, temperate steppe; MM, mountain meadow; GE, grazing exclusion plots; FG, freely grazed plots.

To elucidate the responses of interspecific interactions within soil bacterial and fungal communities to grazing exclusion across different grassland types, we constructed microbial co-occurrence networks based on Spearman correlations between soil microbial OTUs (Figure 2a,b). Concurrently, we calculated key topological parameters (nodes, edges, average degree, average clustering coefficient, and modularity) to quantify the complexity of the soil microbial networks in each grassland type. The results showed that regardless of grazing exclusion, the soil microbial networks across all grassland types were characterized by predominantly positive associations. For soil bacteria, grazing exclusion increased the proportion of positive correlations in the networks of the temperate desert (TD) and mountain meadow (MM) from 63.62% and 53.53% to 70.32% and 75.06%, respectively. Conversely, in the temperate steppe (TS), the proportion of positive correlations decreased from 91.22% to 83.11% (Figure 2a). Regarding soil fungal networks, the proportion of positive correlations remained unchanged in TD, decreased by 2.28% in TS, and increased by 1.82% in MM following grazing exclusion (Figure 2b).

The topological parameters of sample-level subnetworks derived from bacterial co-occurrence networks exhibited divergent responses to grazing exclusion across the three grassland types (Figure 2c–g). In TD, grazing exclusion significantly increased the average degree but significantly decreased modularity (both *p* < 0.01). In TS, only the number of nodes increased significantly (*p* < 0.05), whereas other topological parameters showed no significant changes. In MM, both the number of edges and average degree decreased significantly (*p* < 0.05), whereas the average clustering coefficient and modularity increased significantly (*p* < 0.05). In contrast, fungal sample-level subnetworks generally showed increasing trends in most topological parameters under grazing exclusion, except for modularity in MM, which decreased (Figure 2c–h). Notably, increases in fungal network parameters in MM were highly significant (*p* < 0.01).

Overall, the integrated microbial network complexity index showed site-dependent responses to grazing exclusion (Figure 2h). Bacterial network complexity exhibited decreasing numerical trends in TD and TS, while no significant difference was detected in MM. In contrast, fungal network complexity increased significantly in TD and MM, whereas the increase in TS was not significant. These contrasting patterns indicate that bacterial and fungal interaction structures responded differently among grassland types under grazing exclusion.

### 3.2. Effects of Grazing Exclusion on Topologically Important Taxa in Soil Microbial Co-Occurrence Networks

**Figure 3:**
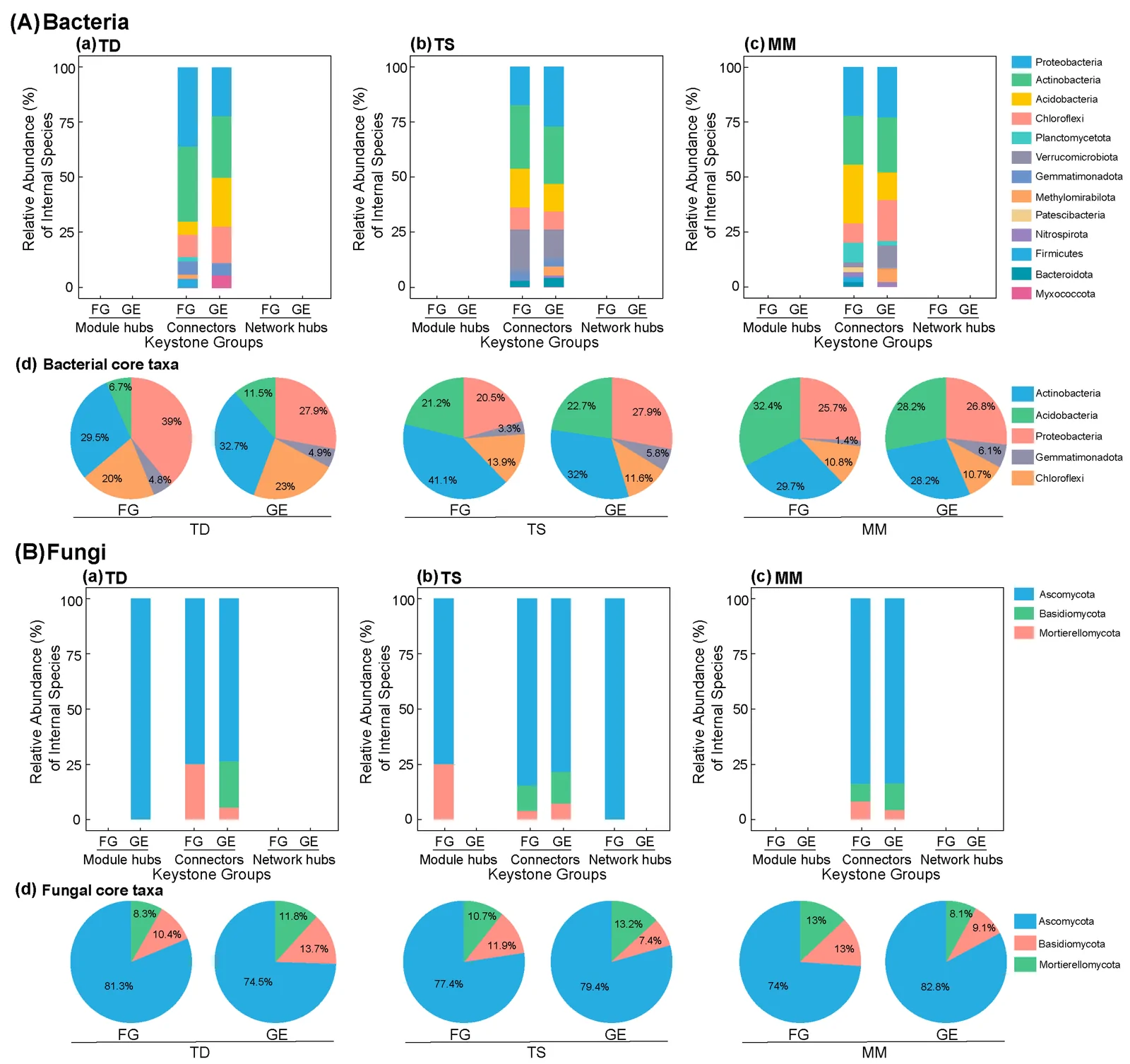
Alterations in keystone taxa of soil microbial co-occurrence networks under grazing exclusion in different grassland types. Note: (**A**) Bacterial communities: (a) Relative abundance of bacterial keystone taxa at the phylum level under freely grazed (FG) and grazing exclusion (GE) treatments in the temperate desert (TD) site. (b) Relative abundance of bacterial keystone taxa in the temperate steppe (TS) site. (c) Relative abundance of bacterial keystone taxa in the mountain meadow (MM) site. (d) Composition of bacterial core taxa at the phylum level under FG and GE treatments across the three sites. (**B**) Fungal communities: (a) Relative abundance of fungal keystone taxa at the phylum level under FG and GE treatments in the TD site. (b) Relative abundance of fungal keystone taxa in the TS site. (c) Relative abundance of fungal keystone taxa in the MM site. (d) Composition of fungal core taxa at the phylum level under FG and GE treatments across the three sites. TD (temperate desert), TS (temperate steppe), MM (mountain meadow), FG (freely grazing plots), GE (grazing exclusion plots).

To characterize the topological roles of microbial taxa within the networks, we performed a node role analysis. Based on within-module connectivity (Zi) and among-module connectivity (Pi), network nodes were categorized into four topological categories: module hubs, network hubs, connectors, and peripherals. Concurrently, Because module hubs, connectors, and network hubs represent topologically important positions within microbial networks, we focused on these three node categories in subsequent analyses (Figure 3A,B). The results showed that after grazing exclusion, topologically important bacterial taxa across all grassland types were primarily distributed within the connector category. Exclusion reduced the number of keystone phyla in the temperate desert (TD) and temperate steppe (TS) but increased their number in the mountain meadow (MM). The dominant bacterial phyla among these topologically important taxa were Proteobacteria, Actinobacteria, Acidobacteria, Chloroflexi, Gemmatimonadota, and Verrucomicrobiota (Figure 3A(a–c)).

Regarding soil fungi, the distribution of topological node roles exhibited divergent patterns across grassland types (Figure 3B(a–c)). In TD, some fungi were categorized as module hubs following exclusion, andthe number of taxa assigned to the connector category increased. In TS, exclusion elevated the proportion of keystone taxa in connectors, whereas taxa previously identified as module hubs and network hubs disappeared. In MM, topologically important fungal taxa were exclusively assigned to the connector category. Across these three grassland types, the dominant fungal phyla among topologically important taxa were Ascomycota, Basidiomycota, and Mortierellomycota.

Furthermore, we analyzed the core microbial communities within the major modules of bacterial and fungal co-occurrence networks under grazing exclusion. In the bacterial networks of TD, the core communities were consistently dominated by Actinobacteria, Proteobacteria, and Chloroflexi. Conversely, the core communities in TS and MM were dominated by Actinobacteria, Proteobacteria, and Acidobacteria (Figure 3A(d)). In contrast, the core microbial communities in fungal networks were uniformly dominated by Ascomycota across all grassland types (Figure 3B(d)).

### 3.3. Effects of Grazing Exclusion on Soil Microbial Community Assembly Processes

**Figure 4:**
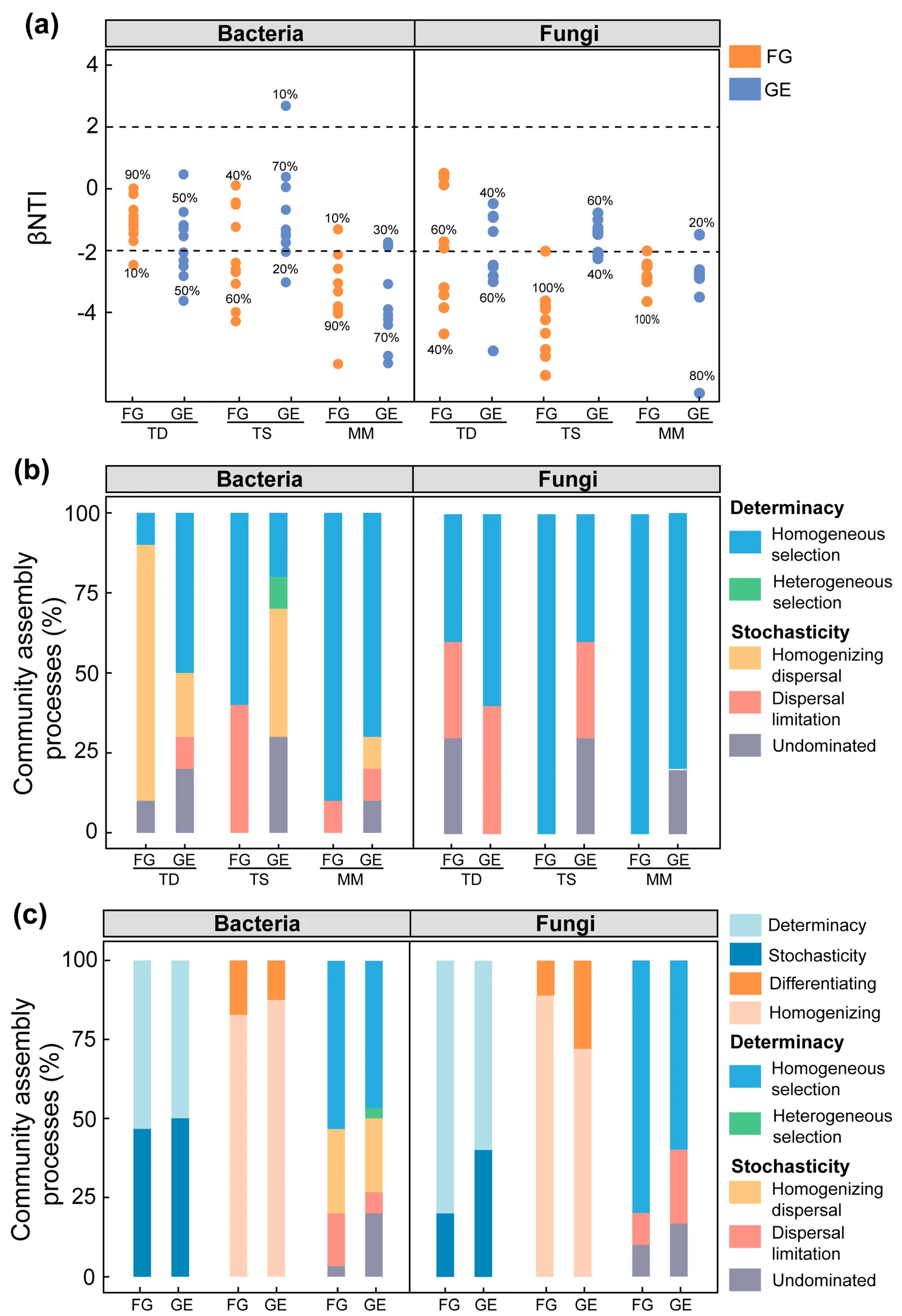
Shifts in soil microbial community assembly processes under grazing exclusion across different grassland types based on pairwise null-model comparisons. Note: (**a**) βNTI values reflecting the relative importance of deterministic versus stochastic processes in bacterial and fungal community assembly under freely grazed (FG) and grazing exclusion (GE) treatments. (**b**) Relative contributions of individual community assembly processes (homogeneous selection, heterogeneous selection, homogenizing dispersal, dispersal limitation, and undominated) for bacterial and fungal communities. (**c**) Relative proportions of broad assembly categories (determinacy, stochasticity, differentiating, and homogenizing) for bacterial and fungal communities. TD (temperate desert), TS (temperate steppe), MM (mountain meadow), FG (freely grazing plots), GE (grazing exclusion plots); Percentages represent the proportions of pairwise comparisons classified into each ecological process category.

Using the β-nearest taxon index (βNTI) threshold (|βNTI| > 2 indicates dominance of deterministic processes; |βNTI| < 2 indicates dominance of stochastic processes), we found consistent shifts in the assembly processes of both bacterial and fungal communities across grassland types following grazing exclusion (Figure 4a). After exclusion, deterministic processes dominated the assembly of both communities in the temperate desert (TD) and mountain meadow (MM), whereas stochastic processes prevailed in the temperate steppe (TS).

Further analysis integrating βNTI with the Bray–Curtis-based Raup–Crick (RCBray) index revealed that homogeneous selection was the primary assembly process for both bacterial and fungal communities in TD and MM, as well as for fungi in TS (Figure 4b). Conversely, bacterial assembly in TS was primarily governed by homogenizing dispersal (Figure 4b).

A quantitative assessment revealed that deterministic and stochastic processes contributed equally (50% each) to bacterial community assembly post-exclusion (Figure 4c). For fungal communities, deterministic processes (60%) outweighed stochastic ones (40%). At the level of specific mechanisms, homogenizing processes (i.e., homogeneous selection and homogenizing dispersal) collectively dominated both communities (Figure 4c). Among these, homogeneous selection was the most prevalent, accounting for 46.67% and 60% of bacterial and fungal assembly, respectively. The secondary processes in bacterial communities were homogenizing dispersal and dispersal limitation (each comprising 23.33%).

### 3.4. Correlation Analysis Between Soil Microbial Network Characteristics, Assembly Processes, and Environmental Factors

**Figure 5:**
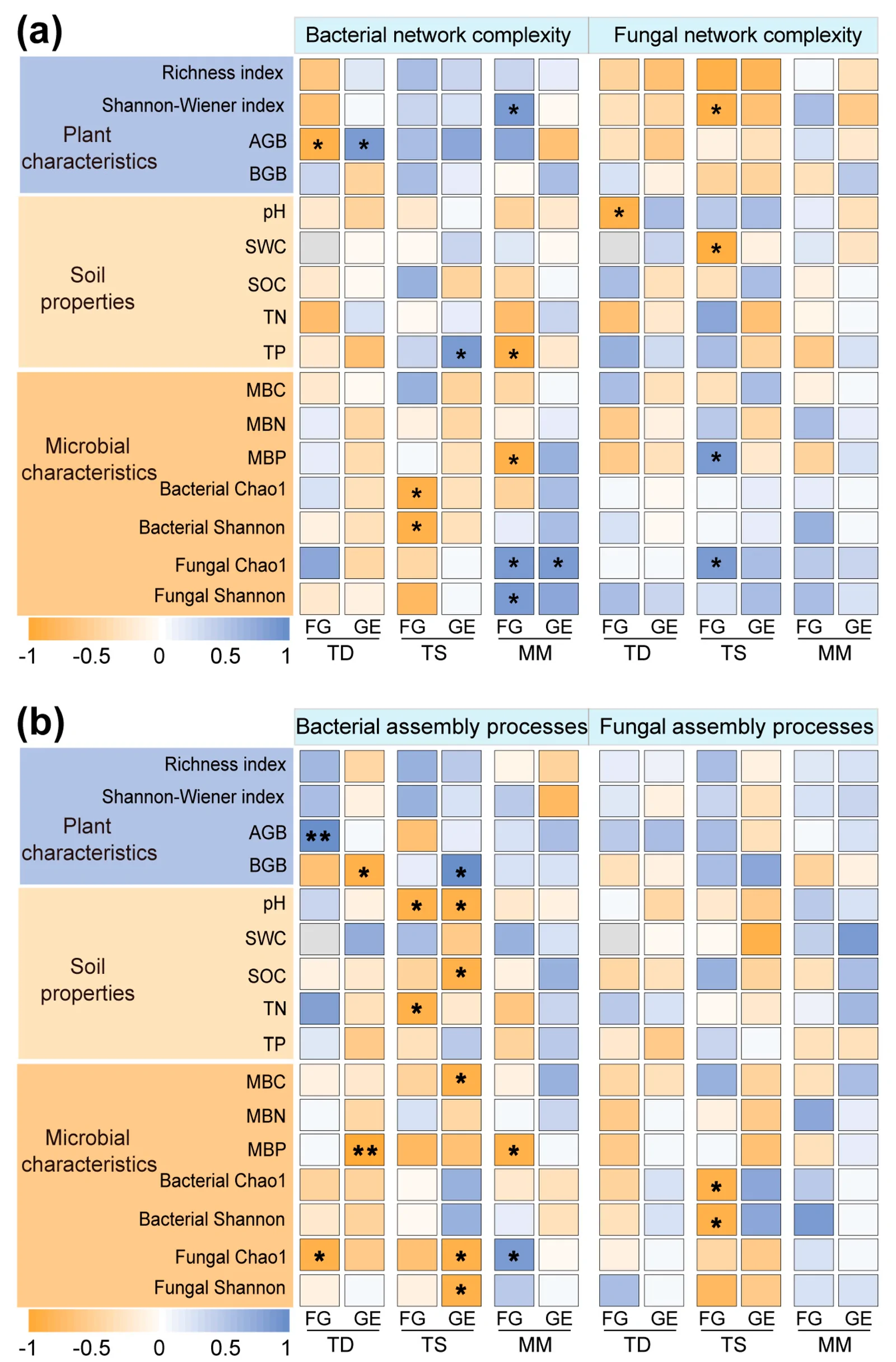
Relationships between environmental variables and microbial network complexity and assembly processes across different grassland types. Note: (**a**) Relationships between environmental variables and bacterial and fungal network complexity. (**b**) Relationships between environmental variables and bacterial and fungal assembly processes. TD (temperate desert), TS (temperate steppe), MM (mountain meadow), FG (freely grazing plots), GE (grazing exclusion plots). Heatmap colors represent Spearman correlation coefficients between environmental variables and microbial network complexity or assembly processes. Asterisks indicate significant correlations within each treatment group (* *p* < 0.05, ** *p* < 0.01). The significance levels represent individual correlations and do not indicate significant differences between correlation coefficients of FG and GE treatments.

To evaluate the associations between environmental variables and microbial network characteristics under different grazing regimes, we performed Spearman correlation analyses to assess the relationships among plant traits, soil physicochemical properties, microbial characteristics, and both microbial network complexity and assembly processes (Figure 5). Grazing exclusion fundamentally altered the correlations between environmental factors and the complexity of bacterial and fungal networks, with distinct patterns across different grassland types. The plant, soil, and microbial variables used in this analysis are detailed in Supplementary Table S1 and Figures S1 and S2.

For bacterial network complexity (Figure 5a), exclusion in the temperate desert (TD) induced a significant positive correlation with above-ground biomass (AGB) (*p* < 0.05). Correlations with plant richness and total nitrogen (TN) showed contrasting directions between grazing exclusion and freely grazed treatments, whereas the associations with microbial biomass nitrogen (MBN), microbial biomass phosphorus (MBP), bacterial Chao1, and fungal Chao1 exhibited different correlation patterns between treatments. In the temperate steppe (TS), exclusion weakened the negative correlations with bacterial Chao1 and Shannon indices while strengthening the positive correlation with total phosphorus (TP) (*p* < 0.05). In the mountain meadow (MM), exclusion weakened the positive correlations with the plant Shannon-Wiener and fungal Shannon indices, as well as the negative correlation with TP. The associations with MBP and bacterial Chao1 showed contrasting directions between treatments, while the positive correlation with the bacterial Shannon index was enhanced. For fungal network complexity, no significant correlations were found across grasslands. However, exclusion reversed the correlation direction for some factors (Figure 5a). In TD, the association with soil pH showed opposite correlation directions between grazing exclusion and freely grazed treatments. In TS, several factors that showed significant correlations under freely grazed conditions, including the plant Shannon–Wiener index, soil water content (SWC), MBP, and fungal Chao1, exhibited different association patterns under grazing exclusion.

Grazing exclusion was associated with differences in the relationships between microbial assembly processes and environmental factors, revealing differentiated patterns (Figure 5b). For bacterial assembly processes, in TD, the significant correlates shifted from AGB and fungal Chao1 to below-ground biomass (BGB) and MBP after exclusion. In TS, post-grazing exclusion assembly showed significant negative correlations with soil pH, SOC, microbial biomass carbon (MBC), fungal Chao1 index, and fungal Shannon index (*p* < 0.05), and a significant positive correlation with BGB (*p* < 0.05). For fungal assembly processes: In TS, exclusion was associated with different correlation patterns with bacterial Chao1 and Shannon indices, while correlations with plant richness, Shannon-Wiener index, and AGB shifted from positive to negative. In MM, most environmental factors showed positive correlations with fungal assembly processes following grazing exclusion.

### 3.5. Effects of Grazing Exclusion on Soil Organic Carbon and Its Associations with Environmental Factors

**Figure 6:**
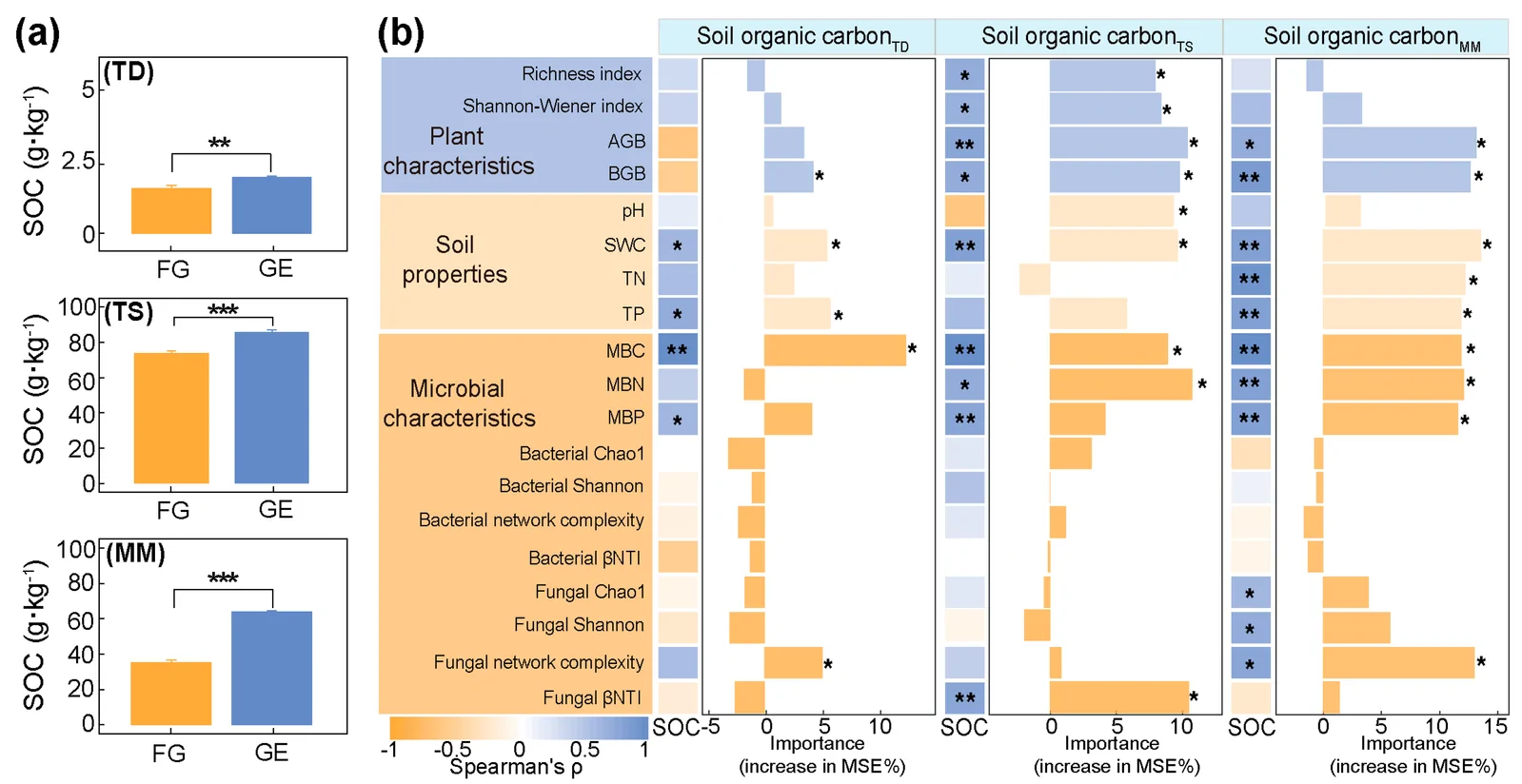
Analysis of grazing exclusion effects on SOC and its environmental associations, showing: (**a**) SOC variation across grassland types, and (**b**) associations between SOC and environmental variables together with relative variable importance estimated by random forest analysis. Note: All data points and error bars in histograms represent mean ± standard error. “*”, “**” and “***” respectively indicated significant (*p* < 0.05), extremely significant (*p* < 0.01) and (*p* < 0.001) differences among different treatments of the same grassland type as determined by independent-sample T tests; TD (temperate desert), TS (temperate steppe), MM (mountain meadow), GE (grazing exclusion plots), FG (freely grazing plots).

Soil organic carbon (SOC) content increased significantly following grazing exclusion across all grassland types, with increments of 24.90%, 16.04%, and 80.65% in the temperate desert (TD), temperate steppe (TS), and mountain meadow (MM), respectively (all *p* < 0.01; Figure 6a).

All environmental variables used in the correlation and random forest analyses are listed in Supplementary Table S1 and Figures S1 and S2. We used an integrated analytical approach to identify key drivers of SOC. First, Spearman correlation analysis assessed associations between SOC and environmental variables (plant traits, soil physicochemical properties, microbial characteristics). Random forest modeling was subsequently used to evaluate the relative importance of environmental variables associated with SOC variation (Figure 6b).

Our analysis revealed that grazing exclusion reshaped the relationships between SOC and environmental factors, with distinct patterns emerging for each grassland type. Specifically, in the temperate desert (TD), SOC showed significant positive correlations with soil water content (SWC), total phosphorus (TP), microbial biomass carbon (MBC), and microbial biomass phosphorus (MBP) (*p* < 0.05). The random forest analysis indicated that below-ground biomass (BGB), SWC, TP, MBC, and fungal network complexity showed relatively higher contributions to SOC variation. In the temperate steppe (TS), SOC was significantly correlated with multiple factors, exhibiting positive correlations with 9 variables, including plant richness, the Shannon-Wiener index, and above-ground biomass (AGB) (*p* < 0.05). The random forest analysis showed relatively higher importance values for several factors, including (such as plant richness, the Shannon-Wiener index, AGB, and BGB) as key predictors. In the mountain meadow (MM), SOC was significantly and positively correlated with 11 environmental factors, including AGB, BGB, and SWC (*p* < 0.05). Among these, Several factors, including AGB, BGB, SWC, and TN, showed relatively higher importance values in explaining SOC variation.

### 3.6. Differentiated Association Pathways of Soil Organic Carbon Variation Across Different Grassland Types Under Grazing Exclusion

**Figure 7:**
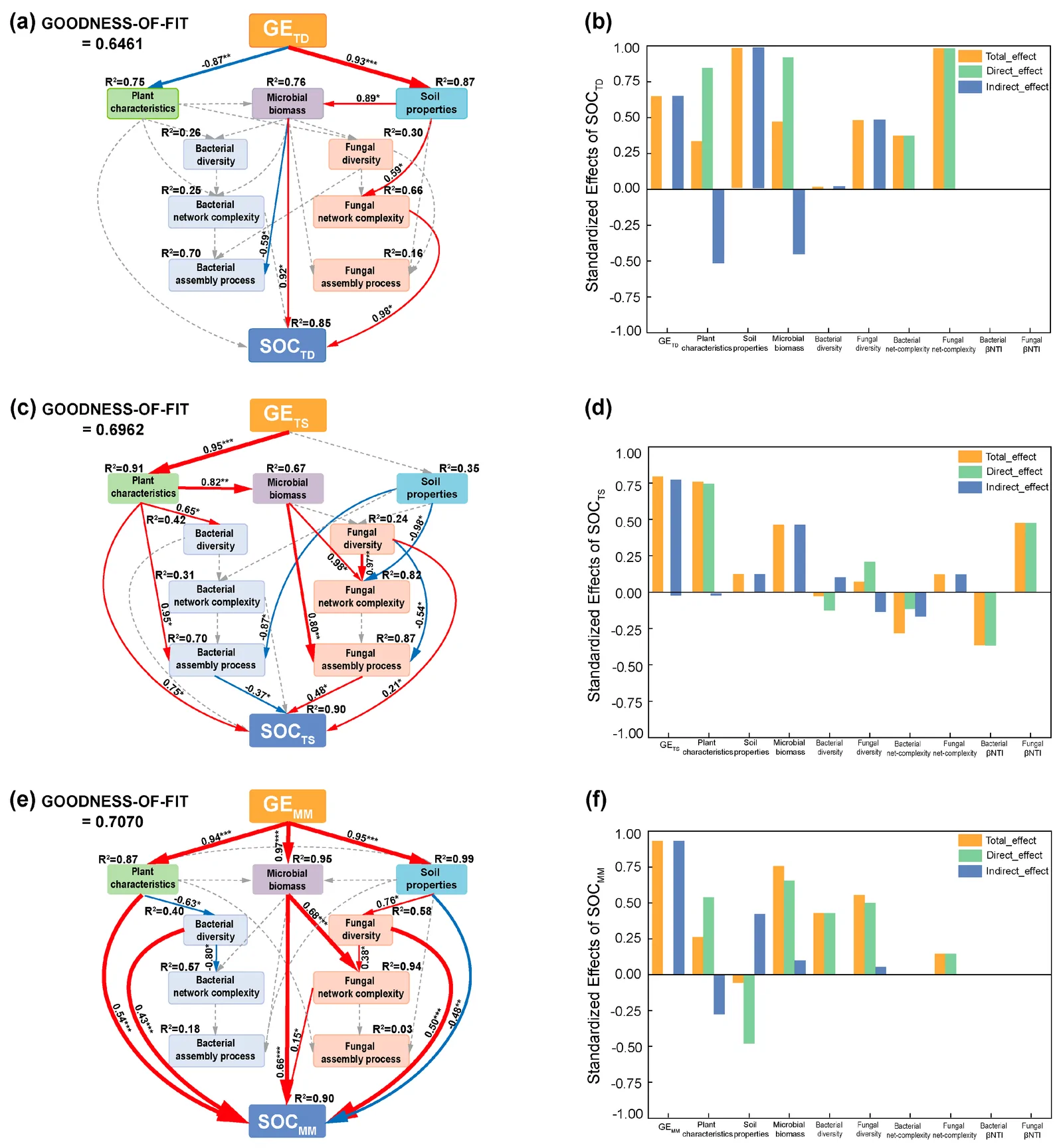
Exploratory PLS-PM analysis illustrating potential statistical association pathways related to soil organic carbon variation under grazing exclusion across grassland types. Note: Potential association pathways related to soil organic carbon (SOC) variation under grazing exclusion across grassland types, as revealed by partial least squares path modeling (PLS-PM). (**a**,**c**,**e**) Exploratory structural equation models for the temperate desert, temperate steppe, and mountain meadow, respectively. (**b**,**d**,**f**) Standardized total association estimates for SOC in each grassland type. Path coefficients are shown on arrows. Solid red and blue arrows represent significant positive and negative associations, respectively, with line thickness proportional to the level of significance (i.e., *** *p* < 0.001, ** *p* < 0.01, * *p* < 0.05). Non-significant associations are shown as thin gray dashed lines of uniform width. R2 values indicate the proportion of variance represented within the model. Standardized path coefficients are shown on arrows. Effect decomposition values represent standardized direct, indirect, and total effects and should not be interpreted as percentage contributions.

To explore the potential pathways associated with SOC variation under grazing exclusion, we employed partial least squares-structural equation modeling (PLS-SEM) using the plant, soil, and microbial dataset provided in Supplementary Table S1. Grazing exclusion showed significant indirect associations with SOC variation across all grasslands, with site-specific total association estimates ranging from 0.6477 to 0.9309 (Figure 7). The associated variables and potential pathways differed among grassland types.

In the temperate desert (TD), the potential association pathway was primarily related to changes in soil properties (pH, SWC, TN, TP), which were associated with SOC variation. On one hand, grazing exclusion significantly altered soil properties, which subsequently increased microbial biomass (MBC, MBN, MBP) and showing a positive indirect association with SOC content (path coefficient = 0.92, *p* < 0.05). On the other hand, grazing exclusion also influenced SOC by modifying soil properties, which then increased fungal network complexity, exerting a positive indirect effect on SOC via this pathway (path coefficient = 0.98, *p* < 0.05). In this exploratory PLS-PM model, microbial biomass and fungal network complexity showed significant direct associations with SOC variation, and the model explained 85% of the variance in SOC content (Figure 7a,b). Specifically, based on effect decomposition (Figure 7b), fungal network complexity showed a positive standardized direct effect (0.983) on SOC variation.

In the temperate steppe (TS), SOC variation was primarily associated with plant traits in the exploratory model (AGB, BGB, richness, Shannon-Wiener index), showing three potential indirect association pathways with SOC. First, grazing exclusion significantly altered plant traits, which directly increased SOC content (path coefficient = 0.75, *p* < 0.05). Second, grazing exclusion regulated plant traits to affect microbial biomass, thereby changing fungal assembly processes and ultimately influencing SOC (path coefficient = 0.48, *p* < 0.05). Additionally, grazing exclusion modified plant traits to influence bacterial community diversity and bacterial assembly processes, subsequently regulating SOC content (path coefficient = −0.37, *p* < 0.05). The main direct regulatory factors for SOC in this grassland were plant traits, bacterial assembly processes, fungal diversity, and fungal assembly processes, together accounting for 90% of the variation represented in the exploratory model, showing a different association pattern from TD. (Figure 7c,d). Specifically, based on effect decomposition (Figure 7d), bacterial network complexity showed a negative standardized direct effect (−0.115) and indirect effect (−0.168) on SOC. Fungal network complexity showed a positive standardized indirect effect (0.121), whereas bacterial assembly processes showed a negative standardized direct effect (−0.366) and fungal assembly processes showed a positive standardized direct effect (0.476).

In the mountain meadow (MM), grazing exclusion exerted more diverse effects by significantly influencing plant traits (AGB, BGB, richness, Shannon-Wiener index), microbial biomass (MBC, MBN, MBP), and soil properties (pH, SWC, TN, TP), which then showed multiple potential association pathways with SOC variation. These pathways mainly included: directly regulating SOC by altering plant community traits (path coefficient = 0.54, *p* < 0.001), and indirectly regulating SOC by changing bacterial diversity (path coefficient = 0.43, *p* < 0.001); directly regulating SOC by affecting microbial biomass (path coefficient = 0.66, *p* < 0.001), and indirectly altering SOC by influencing fungal network complexity (path coefficient = 0.15, *p* < 0.05); furthermore, grazing exclusion showed a direct association with SOC through soil properties (path coefficient = −0.48, *p* < 0.01), and indirectly affected fungal diversity through soil properties to regulate SOC at multiple levels (path coefficient = 0.50, *p* < 0.001). Variables showing significant direct associations included plant traits, microbial biomass, soil properties, bacterial diversity, fungal diversity, and fungal network complexity, collectively explaining 90% of the variation in SOC content (Figure 7e,f). Specifically, based on effect decomposition (Figure 7f), bacterial diversity showed a positive standardized direct effect (0.430) on SOC. Fungal diversity showed positive standardized direct and indirect effects (0.499 and 0.056, respectively), while fungal network complexity showed a positive standardized direct effect (0.147), consistent with the corresponding path coefficient.

## 4. Discussion

### 4.1. Divergent Restructuring of Soil Microbial Networks Under Grazing Exclusion

Soil microorganisms coexist within complex communities and exhibit correlation-based co-occurrence patterns, which may reflect aspects of microbial community organization and ecosystem ecological processes [37,38]. In this study, positive correlations dominated bacterial and fungal co-occurrence networks across all three investigated sites, regardless of grazing exclusion treatment (Figure 2). This pattern is consistent with the stress gradient hypothesis, which suggests that microbial communities under environmental stress may exhibit stronger positive associations among taxa, reflecting potential ecological relationships under environmental constraints [39,40].

Grazing exclusion was associated with distinct changes in bacterial and fungal network properties across the three investigated sites. Overall, bacterial network complexity tended to decrease after grazing exclusion, whereas fungal network complexity increased and exhibited greater connectivity characteristics (Figure 2c–h). These contrasting responses may reflect differences in the ecological strategies of bacteria and fungi. Bacteria, particularly rapidly responding taxa such as members of Proteobacteria, are often closely associated with labile carbon sources and short-term resource fluctuations, including inputs derived from grazing animals and plant activity [41,42]. Therefore, the removal of grazing disturbance may alter the quantity and quality of available resources, potentially contributing to changes in bacterial community co-occurrence patterns and reduced network complexity. In contrast, fungi, especially members of Ascomycota, are important decomposers of complex organic substrates [43]. The increased vegetation biomass and litter inputs following grazing exclusion may provide additional substrates for fungal growth and co-occurrence associations, which could be associated with increased fungal network complexity. However, these network changes should be interpreted as shifts in microbial interaction patterns rather than direct evidence of altered ecosystem stability or functionality.

The responses of microbial networks varied among the three investigated sites, highlighting the potential influence of local environmental conditions on microbial responses to grazing exclusion. For example, bacterial network modularity showed contrasting responses between the arid TD site and the relatively humid MM site (Figure 2), suggesting that differences in initial soil and vegetation conditions may contribute to variation in microbial network responses. Despite differences in network topology, core microbial taxa remained relatively stable after grazing exclusion. Bacterial networks were consistently dominated by Actinobacteria, Proteobacteria, and Acidobacteria, while fungal networks were primarily characterized by Ascomycota (Figure 3A,B and Figure S1). These dominant taxa are widely recognized as important participants in soil carbon and nutrient cycling processes [30,40,44]. Although their overall taxonomic dominance remained stable, grazing exclusion altered the topological positions of some topologically important taxa within microbial networks, such as changes in module hub and connector roles (Figure 3). Such changes may indicate adjustments in microbial interaction patterns and potential ecological roles under altered environmental conditions.

Grazing exclusion was also associated with changes in the relationships between microbial network characteristics and environmental variables, and these relationships differed among the investigated sites (Figure 5). For bacterial networks, associations with environmental variables changed after exclusion, such as the shift from negative to positive associations between bacterial network properties and aboveground biomass in the TD site. This suggests that vegetation recovery and soil environmental changes may be linked to altered bacterial interaction patterns. In contrast, fungal network complexity showed relatively weak relationships with individual measured environmental variables, indicating that fungal network organization may be related to multiple ecological processes rather than a single environmental factor. The concurrent changes in fungal network properties and microbial assembly processes further suggest that variations in fungal interactions may be associated with shifts in community assembly patterns under grazing exclusion.

An important question is why SOC content was higher despite the site-dependent responses of bacterial network complexity. The observed changes in bacterial network complexity do not necessarily indicate reduced SOC retention, because SOC variation reflects the balance between carbon inputs and microbial carbon processing over time. First, increased fungal network complexity in some sites (particularly TD and MM) may represent one potential ecological characteristic associated with higher SOC content, as fungi, particularly Ascomycota, play important roles in processing complex plant-derived organic matter accumulated after grazing exclusion. Previous studies have also suggested that fungal network characteristics can be associated with potential ecological processes [10,19]. Second, grazing exclusion altered plant biomass differently among grassland types (Table S1). Increased aboveground and belowground biomass in TS and MM may have enhanced carbon inputs through plant litter and root-derived carbon, whereas biomass responses were not consistent in TD. These site-specific vegetation responses may have contributed to the higher SOC content observed after grazing exclusion. Third, changes in bacterial network organization may reflect shifts in microbial resource utilization strategies rather than directly determining SOC variation. Consistent with this interpretation, our SEM results (Figure 7) showed that fungal network complexity, rather than bacterial network complexity, exhibited significant associations with SOC at the TD and MM sites. Therefore, SOC variation under grazing exclusion likely reflects the combined associations among plant inputs, soil conditions, and microbial ecological characteristics rather than the effect of a single microbial network property.

The divergent bacterial and fungal network responses among the three investigated sites may be related to differences in environmental conditions. At the TD site, resource limitation and arid conditions were associated with stronger changes in microbial assembly and network properties. At the MM site, favorable environmental conditions coincided with increased fungal network complexity and stronger plant–soil–microbial associations. At the TS site, microbial network responses were more closely associated with vegetation recovery. These patterns suggest that microbial network responses to grazing exclusion are context dependent rather than following a universal trajectory.

### 4.2. Grazing Exclusion Alters the Balance Between Deterministic and Stochastic Assembly Processes

Soil microbial community assembly processes showed distinct responses to grazing exclusion among the three investigated sites (Figure 4). At the TD and MM sites, both bacterial and fungal communities exhibited increased contributions of deterministic processes, particularly homogeneous selection, following grazing exclusion. In contrast, at the TS site, bacterial communities showed an increased contribution of stochastic processes, especially homogenizing dispersal, whereas fungal communities remained primarily associated with homogeneous selection despite a relatively high contribution of stochastic processes. These contrasting patterns suggest that grazing exclusion may influence the relative balance between deterministic and stochastic assembly processes depending on local environmental conditions.

The increased contribution of homogeneous selection at the TD and MM sites may be related to changes in environmental conditions following grazing exclusion. The removal of grazing disturbance may alter vegetation structure and soil microhabitat conditions, potentially affecting the strength of environmental filtering processes [45]. This interpretation is consistent with previous studies showing that homogeneous selection tends to increase under relatively consistent resource conditions [34,46]. Supporting this possibility, bacterial assembly processes at the TD site showed stronger associations with belowground biomass (BGB) and microbial biomass phosphorus (MBP) after grazing exclusion, suggesting that changes in resource availability may be related to shifts in bacterial community assembly patterns [47,48].

In contrast, bacterial communities at the TS site exhibited an increased contribution of homogenizing dispersal after grazing exclusion. This pattern may be associated with enhanced habitat connectivity and microbial dispersal under conditions of rapid vegetation recovery. When dispersal processes become relatively strong, stochastic processes can contribute substantially to community assembly [49,50]. Although bacterial assembly patterns at the TS site were correlated with several soil properties, these environmental variables may have been insufficient to explain the observed assembly patterns relative to dispersal-related processes. In comparison, fungal communities at the TS site maintained a dominant contribution of homogeneous selection despite increased stochasticity. This difference may reflect contrasting ecological characteristics between bacteria and fungi, including differences in resource acquisition strategies and dispersal capacities [51,52,53]. The contrasting assembly responses between bacterial and fungal communities highlight the importance of considering microbial groups separately when evaluating management-induced ecological changes.

The differences in assembly responses among the three investigated sites may reflect contrasting environmental contexts. At the arid TD site, limited water availability and resource constraints may strengthen environmental filtering, whereas at the TS site, rapid vegetation recovery may increase dispersal opportunities, particularly for bacterial communities. The MM site showed stronger deterministic signals under relatively favorable and less disturbed conditions. These patterns indicate that the relative contributions of deterministic and stochastic processes may vary among ecosystems depending on local environmental conditions and disturbance history.

From a broader ecological perspective, these site-specific patterns reflect differences in the relative importance of environmental filtering and dispersal processes under different resource conditions. In the TD site, grazing exclusion was associated with stronger deterministic assembly patterns under arid conditions, whereas in the TS site, bacterial communities showed greater stochastic signatures potentially associated with enhanced dispersal. Such variation is consistent with theoretical expectations that environmental gradients can modify the relative contributions of deterministic and stochastic processes in microbial community assembly [54,55,56,57]. However, these interpretations should be considered as ecological explanations for observed patterns rather than direct evidence of causal mechanisms.

Changes in microbial assembly processes may also be associated with SOC variation under grazing exclusion. Homogeneous selection may reflect stronger environmental filtering on microbial communities, which could be associated with differences in microbial ecological characteristics. At the TD and MM sites, stronger deterministic assembly patterns were accompanied by increased SOC content, which may be related to the combined effects of plant carbon inputs, soil conditions, and microbial community characteristics. At the TS site, bacterial assembly processes dominated by homogenizing dispersal showed a negative association with SOC, whereas fungal assembly processes dominated by homogeneous selection showed a positive association with SOC (Figure 7d). These results suggest that bacterial and fungal assembly processes may have different relationships with SOC variation, and that the net SOC response likely reflects interactions among microbial properties, vegetation recovery, and soil environmental conditions rather than the effect of assembly processes alone.

Across the three investigated sites, fungal communities generally showed a higher relative contribution of deterministic processes than bacterial communities following grazing exclusion. This pattern may be related to differences in the ecological strategies of fungi and bacteria and their contrasting responses to grazing exclusion (Figure 2). Overall, our results indicate that grazing exclusion is associated with site-specific shifts in microbial community assembly processes, with deterministic processes becoming more prominent at TD and MM sites and stochastic processes increasing for bacterial communities at the TS site. These findings highlight that microbial responses to grazing exclusion depend on local environmental contexts and provide insights into how restoration practices may generate different belowground ecological outcomes under contrasting grassland conditions.

### 4.3. Microbial Network Characteristics and Assembly Processes Are Associated with Site-Specific SOC Variation Under Grazing Exclusion

Our study showed that grazing exclusion was associated with increased surface SOC content at the three investigated sites, with increases of 24.90%, 16.04%, and 80.65% in the temperate desert (TD), temperate steppe (TS), and mountain meadow (MM), respectively (Figure 6a). However, the magnitude of these differences varied considerably among sites, highlighting the context-dependent responses of SOC to grazing exclusion. Furthermore, the ecological factors associated with SOC variation differed among the three sites. Initial correlation analysis and random forest modeling identified distinct key predictors associated with SOC differences between grazing exclusion and freely grazed plots (Figure 6b). At the TD site, SOC was closely associated with soil properties (SWC, TP, MBC) and fungal network complexity. At the TS site, SOC variation was more strongly related to plant community characteristics (e.g., richness, Shannon index, and AGB). At the MM site, plant, soil, and microbial factors were simultaneously associated with SOC variation. These differences suggest that the ecological components associated with grazing exclusion responses vary depending on local environmental conditions [56,58].

To further examine the statistical relationships among these ecological components and SOC, we employed partial least squares structural equation modeling (PLS-SEM). The models provided an exploratory assessment of potential associations among environmental variables and SOC variation and identified site-specific association patterns (Figure 7). Across these pathways, microbial network characteristics and assembly processes showed potential intermediate associations between plant/soil conditions and SOC variation, although the strength of these relationships varied among sites and some pathways were not statistically significant [10].

At the TD site, where soil conditions represent an important ecological constraint, SOC variation was mainly associated with a soil-related pathway (Figure 7a,b). Grazing exclusion was associated with improved soil properties (e.g., SWC and TP), which were further related to microbial biomass and fungal network complexity. This pattern is consistent with our network analysis showing increased fungal network complexity after exclusion at the TD site, together with stronger positive connectivity among fungal taxa. Such changes may indicate enhanced microbial co-occurrence patterns under improved soil conditions, although direct functional consequences require further validation [10,39]. Within the exploratory PLS-SEM framework, microbial biomass and fungal network complexity were associated with SOC variation and accounted for 85% of the variance represented in the model. This pattern was also consistent with the observed increase in homogeneous selection in microbial assembly at this site, suggesting that changes in soil conditions were associated with shifts in microbial ecological characteristics [34,46]. Therefore, in this resource-limited site, soil improvement following grazing exclusion appeared to be closely associated with changes in microbial biomass and fungal network properties, which together corresponded with SOC variation.

At the TS site, vegetation recovery represented an important ecological component associated with SOC variation after grazing exclusion (Figure 7c,d). Plant traits showed the strongest contribution in the random forest model and represented central variables in the SEM pathways. Within this pathway, microbial assembly processes exhibited additional associations with SOC variation. Following exclusion, bacterial communities showed an increased contribution of homogenizing dispersal, which showed a negative association with SOC in the SEM model. One possible explanation is that enhanced vegetation development and associated changes in habitat connectivity may increase microbial dispersal opportunities, potentially introducing taxa with different ecological characteristics [50,56]. However, because direct measurements of microbial functional traits and carbon transformation processes were not conducted, this interpretation requires further investigation.

At the 12-year experimental timescale, the positive association between vegetation recovery and SOC was stronger than the negative relationship associated with bacterial stochastic assembly, which coincided with higher SOC content in exclusion plots. Meanwhile, fungal assembly processes also showed important associations within this pathway. The SEM model indicated that plant traits were positively related to microbial biomass, which was further associated with fungal assembly characteristics dominated by homogeneous selection, and this pathway showed a positive relationship with SOC (path coefficient = 0.48). The changes in microbial diversity following vegetation recovery (Figure S2A,B) suggest that microbial communities responded to vegetation changes, although the responses differed between bacterial and fungal communities. In TS, bacterial diversity showed increases in some diversity indices, whereas fungal diversity did not show significant changes. However, bacterial diversity increases were not accompanied by higher network complexity, potentially reflecting differences in the ecological organization of bacterial and fungal communities. In contrast, increased fungal diversity together with deterministic assembly patterns may have been associated with greater fungal network complexity and SOC variation.

Overall, the TS site demonstrates that even when vegetation recovery represents the dominant ecological component associated with SOC responses, microbial assembly patterns may contribute additional variation through different relationships with SOC. Thus, microbial assembly processes may represent an ecological component associated with the relationship between vegetation changes and SOC responses rather than an independent determinant. These findings indicate that long-term SOC responses may reflect interactions among plant inputs, microbial community organization, and environmental conditions [59].

In the structurally complex mountain meadow (MM) site, multiple ecosystem components were simultaneously associated with SOC variation, representing a multi-component association pattern (Figure 7e,f). The factors identified by the random forest model were also represented in the exploratory SEM analysis, where plant traits, soil properties, and microbial biomass showed significant relationships with SOC variation. These ecological components were associated with multiple microbial attributes, including bacterial and fungal diversity and fungal network complexity. In this site, microbial responses showed considerable complexity: although bacterial network complexity slightly decreased after grazing exclusion, core bacterial taxa (e.g., Actinobacteria and Proteobacteria) remained relatively stable [30]. In contrast, fungal network complexity increased, accompanied by a strengthened contribution of core Ascomycota taxa, potentially reflecting increased resource availability associated with vegetation recovery and litter inputs [43,44]. These patterns suggest that different microbial groups may respond differently to altered environmental conditions, and that their combined responses were associated with SOC variation. The SEM model accounted for 90% of the variance represented in the model, suggesting that the selected plant–soil–microbial variables captured major statistical associations with SOC variation at the MM site [60]. However, further experimental evidence is required to determine whether microbial community reorganization directly contributes to SOC stabilization.

Although the ecological factors associated with SOC variation differed among the three investigated sites (soil-associated pathway in TD, plant-associated pathway in TS, and multi-component association pattern in MM), a common pattern emerged: grazing exclusion was consistently associated with changes in microbial ecological characteristics, particularly fungal network complexity and community assembly processes, and these changes showed statistical relationships with SOC variation. At the TD and MM sites, increased contributions of deterministic assembly processes and enhanced fungal network complexity coincided with positive associations with SOC in the SEM models. At the TS site, although bacterial communities showed stronger stochastic assembly patterns, fungal assembly characteristics and vegetation-related variables were positively associated with SOC variation. These results support the conceptual framework proposed in the Introduction that grazing exclusion may be associated with changes in environmental conditions, microbial ecological properties, and SOC responses. However, this framework should be interpreted as a statistical association pathway rather than a confirmed causal cascade. Although mediation analysis in SEM provides information about potential indirect relationships, direct biogeochemical evidence (e.g., microbial necromass production and carbon use efficiency) is still required to clarify the mechanisms underlying SOC stabilization.

From a management perspective, our results suggest that grazing exclusion responses may vary depending on local environmental contexts rather than following a universal pattern. We therefore propose a site-diagnosis-based perspective for interpreting restoration outcomes. In soil-limited systems such as TD, soil improvement was closely associated with SOC responses; in vegetation-responsive systems such as TS, plant recovery represented an important ecological component; whereas in more tightly coupled systems such as MM, simultaneous plant–soil–microbial changes were associated with SOC variation.

However, these interpretations are based on a single 12-year experiment conducted at three investigated sites on the northern slope of the Tianshan Mountains. Therefore, broader spatial and temporal gradients are required to evaluate whether these site-specific patterns can be generalized to other grassland ecosystems. Moreover, although our study identified consistent statistical associations between microbial ecological characteristics and SOC variation, we did not directly quantify key biogeochemical processes, including microbial necromass formation, mineral-associated organic matter formation, or carbon use efficiency. Therefore, microbial network characteristics and community assembly processes should be considered potential ecological indicators or mediating factors associated with SOC responses rather than direct biochemical mechanisms of SOC stabilization. Future studies integrating microbial functional traits, carbon flux measurements, and molecular-level carbon stabilization processes will be necessary to further resolve the mechanisms underlying SOC responses to grazing exclusion.

## 5. Conclusions

Our 12-year study indicates that grazing exclusion was associated with significant increases in surface SOC content (16.04–80.65%) across the three investigated sites representing temperate desert, temperate steppe, and mountain meadow ecosystems. However, this common pattern was accompanied by divergent site-specific statistical pathways: soil-associated patterns in the temperate desert site, plant-associated patterns in the temperate steppe site, and multi-component associations in the mountain meadow site. Across all investigated sites, changes in microbial ecological properties—particularly fungal network complexity and community assembly processes—were consistently associated with site-specific environmental changes and SOC variation, suggesting that these microbial characteristics may serve as important ecological indicators of SOC responses. Nevertheless, the absence of direct measurements of microbial necromass contribution, SOC fractions, and microbial carbon-use efficiency limits our ability to determine the specific biochemical processes underlying SOC stabilization.

Future research should prioritize (1) disentangling the temporal dynamics of microbial networks and assembly processes following grazing exclusion; (2) testing the general applicability of these observed patterns across broader geographical and ecological gradients; and (3) integrating microbial ecological indicators with direct measurements of carbon transformation processes to improve predictive understanding of grassland carbon dynamics.

## Data Availability

The raw amplicon sequencing data supporting this study have been deposited in the NCBI BioProject under accession number PRJNA1445889 and are publicly available. Other plant and soil physicochemical data are available from the corresponding author upon reasonable request.

## Supplementary Materials

The following supporting information can be downloaded at: https://www.mdpi.com/article/10.3390/microorganisms14092023/s1, Table S1: Effects of enclosure on plant, soil, and microbial characteristics of different grassland types; Figure S1: Effects of enclosure on microbial community structure at phylum level of different grassland types; Figure S2: Effects of enclosure on microbial community diversity of different grassland types.

## References

[1] McDonald S.E., Badgery W., Clarendon S., Orgill S., Sinclair K., Meyer R., Butchart D.B., Eckard R., Rowlings D., Grace P. (2023). Grazing management for soil carbon in Australia: A review. J. Environ. Manag..

[2] Bai Y.F., Cotrufo M.F. (2022). Grassland soil carbon sequestration: Current understanding, challenges, and solutions. Science.

[3] Li Y., Buckeridge K., Wang B., Huang Q., Liu C., Chen Y., Rocha A.V.S., An S. (2025). Grazing exclusion enhanced the capability of soil microorganisms to access photosynthetic carbon in Loess Plateau grassland. Soil Biol. Biochem..

[4] Qu Q., Deng L., Shangguan Z., Sun J., He J., Wang K., Zhou Z., Li J., Peñuelas J. (2024). Belowground C sequestrations response to grazing exclusion in global grasslands: Dynamics and mechanisms. Agric. Ecosyst. Environ..

[5] Liu H., Gong Y., Li Y., Liu S., Yu Z., Zhao R. (2023). Does grazing exclusion enhance grassland restoration? Evidence from northern China. Ecol. Indic..

[6] Li W., Cao W., Wang J., Li X., Xu C., Shi S. (2017). Effects of grazing regime on vegetation structure, productivity, soil quality, carbon and nitrogen storage of alpine meadow on the Qinghai-Tibetan Plateau. Ecol. Eng..

[7] Aynekulu E., Mekuria W., Tsegaye D., Feyissa K., Angassa A., de Leeuw J., Shepherd K. (2017). Long-term livestock exclosure did not affect soil carbon in southern ethiopian rangelands. Geoderma.

[8] Wang S., Zhang S., Lin X., Li X., Li R., Zhao X., Liu M. (2022). Response of soil water and carbon storage to short-term grazing prohibition in arid and semi-arid grasslands of China. J. Arid Environ..

[9] Wang H., Li Y., He Y., Chen H.Y., Liu X., Gao Y., Zhu W., Xu J., Li Y., Chen Z. (2023). Grazing exclusion facilitates more rapid ecosystem carbon sequestration of degraded grasslands in humid than in arid regions. Agric. Ecosyst. Environ..

[10] Wagg C., Schlaeppi K., Banerjee S., Kuramae E.E., Van Der Heijden M.G.A. (2019). Fungal-bacterial diversity and microbiome complexity predict ecosystem functioning. Nat. Commun..

[11] Wang X., Hou Y., Li H., Li Z., Zhang J., Bao T., Chao L., Minggagud H., Wang L., Liang C. (2024). Network complexity and community composition of key bacterial functional groups promote ecosystem multifunctionality in three temperate steppes of Inner Mongolia. Plant Soil.

[12] Zhang C., Liu G., Song Z., Wang J., Guo L. (2018). Interactions of soil bacteria and fungi with plants during long-term grazing exclusion in semiarid grasslands. Soil Biol. Biochem..

[13] Lavallee J.M., Chomel M., Segura N.A., de Castro F., Goodall T., Magilton M., Rhymes J.M., Delgado-Baquerizo M., Griffiths R.I., Baggs E.M. (2024). Land management shapes drought responses of dominant soil microbial taxa across grasslands. Nat. Commun..

[14] Chen W., Wang J., Chen X., Meng Z., Xu R., Duoji D., Zhang J., He J., Wang Z., Chen J. (2022). Soil microbial network complexity predicts ecosystem function along elevation gradients on the tibetan plateau. Soil Biol. Biochem..

[15] Ding L., Tian L., Li J., Zhang Y., Wang M., Wang P. (2023). Grazing lowers soil multifunctionality but boosts soil microbial network complexity and stability in a subtropical grassland of China. Front. Microbiol..

[16] Yang X., Yun X., Zhang W., Struik P.C., Jiang S., Tu X., Jin K., Wang Z. (2025). Contrasting responses of surface and subsurface soil microbiome to ecological restoration in two types of steppe because of different changes in plant and soil properties. Appl. Soil Ecol..

[17] Wang F., Li Z., Fu B., Lü Y., Liu G., Wang D., Wu X. (2022). Short-term grazing exclusion alters soil bacterial co-occurrence patterns rather than community diversity or composition in temperate grasslands. Front. Microbiol..

[18] Khatri-Chhetri U., Banerjee S., Thompson K.A., Quideau S.A., Boyce M.S., Bork E.W., Carlyle C.N. (2024). Cattle grazing management affects soil microbial diversity and community network complexity in the Northern Great Plains. Sci. Total Environ..

[19] Mi W., Meng R., Ren W., Yuan T., Liu Y., Han H., Liang J., Zhang J. (2026). Artificial restoration improved the complexity of the soil microbial co-occurrence network and the resistance of microbial communities to environmental changes in degraded sandy grassland. Agric. Ecosyst. Environ..

[20] Zhou J., Ning D. (2017). Stochastic community assembly: Does it matter in microbial ecology?. Microbiol. Mol. Biol. Rev..

[21] Ning D., Yuan M., Wu L., Zhang Y., Guo X., Zhou X., Yang Y., Arkin A.P., Firestone M.K., Zhou J. (2020). A quantitative framework reveals ecological drivers of grassland microbial community assembly in response to warming. Nat. Commun..

[22] Jiao S., Zhang B., Zhang G., Chen W., Wei G. (2021). Stochastic community assembly decreases soil fungal richness in arid ecosystems. Mol. Ecol..

[23] Hu Y., Liu W., Chang J., Fan Y., Hou S., Zhang Z., Su X., Bahram M., Wang S. (2024). Grazing exclusion-induced alterations of soil microbial biogeographic pattern and co-occurrence network across a Tibetan elevation gradient. Agric. Ecosyst. Environ..

[24] Graham E.B., Stegen J.C. (2017). Dispersal-based microbial community assembly decreases biogeochemical function. Processes.

[25] Liu H., Sun Z., Dong Y., Yang H., He P., Yu B., Ye H., Li S., Zhou L. (2022). Precipitation drives the accumulation of soil organic carbon in the sandy desert of the Junggar Basin, Northwest China. Ecol. Indic..

[26] Bao S.D. (2000). Soil and Agricultural Chemistry Analysis.

[27] Vance E.D., Brookes P.C., Jenkinson D.S. (1987). An extraction method for measuring soil microbial biomass C. Soil Biol. Biochem..

[28] Brookes P., Landman A., Pruden G., Jenkinson D. (1985). Chloroform fumigation and the release of soil nitrogen: A rapid direct extraction method to measure microbial biomass nitrogen in soil. Soil Biol. Biochem..

[29] Yao Z., Huang C., Hu H., Wang T., Li Y., Sun X., Adl S., Zhu B. (2024). High trophic level organisms and the complexity of soil micro-food webs at aggregate scale regulate carbon accumulation in cropland soils. Agric. Ecosyst. Environ..

[30] Banerjee S., Schlaeppi K., van der Heijden M.G. (2018). Keystone taxa as drivers of microbiome structure and functioning. Nat. Rev. Microbiol..

[31] Yuan M.M., Guo X., Wu L., Zhang Y., Xiao N., Ning D., Shi Z., Zhou X., Wu L., Yang Y. (2021). Climate warming enhances microbial network complexity and stability. Nat. Clim. Change.

[32] Stegen J.C., Lin X.J., Konopka A.E., Fredrickson J.K. (2012). Stochastic and deterministic assembly processes in subsurface microbial communities. ISME J..

[33] Stegen J.C., Lin X., Fredrickson J.K., Chen X., Kennedy D.W., Murray C.J., Rockhold M.L., Konopka A. (2013). Quantifying community assembly processes and identifying features that impose them. ISME J..

[34] Dini-Andreote F., Stegen J.C., van Elsas J.D., Salles J.F. (2015). Disentangling mechanisms that mediate the balance between stochastic and deterministic processes in microbial succession. Proc. Natl. Acad. Sci. USA.

[35] Tripathi B.M., Stegen J.C., Kim M., Dong K., Adams J.M., Lee Y.K. (2018). Soil pH mediates the balance between stochastic and deterministic assembly of bacteria. ISME J..

[36] Wu M.-H., Xue K., Wei P.-J., Jia Y.-L., Zhang Y., Chen S.-Y. (2022). Soil microbial distribution and assembly are related to vegetation biomass in the alpine permafrost regions of the Qinghai-Tibet Plateau. Sci. Total Environ..

[37] Gao C., Xu L., Montoya L., Madera M., Hollingsworth J., Chen L., Purdom E., Singan V., Vogel J., Hutmacher R.B. (2022). Co-occurrence networks reveal more complexity than community composition in resistance and resilience of microbial communities. Nat. Commun..

[38] Zhou J., Wang P., Wei L., Zhang J., Li X., Huang N., Liu G., Zou K., Fan R., Liu L. (2025). Grazing increases the complexity of networks and ecological stochastic processes of mycorrhizal fungi. J. Environ. Manag..

[39] Hernandez D.J., David A.S., Menges E.S., A Searcy C., E Afkhami M. (2021). Environmental stress destabilizes microbial networks. ISME J..

[40] Zhang Y., Niu D., Li Q., Liu H., Wang Y., Xu J., Du B., Guo D., Liu Y., Fu H. (2025). Nonlinear response of soil microbial network complexity to long-term nitrogen addition in a semiarid grassland: Implications for soil carbon processes. Agric. Ecosyst. Environ..

[41] Wang J., Liu G., Zhang C., Wang G., Fang L., Cui Y. (2019). Higher temporal turnover of soil fungi than bacteria during long-term secondary succession in a semiarid abandoned farmland. Soil Tillage Res..

[42] Zhang X., Feng Q., Cao J., Liu W., Qin Y., Zhu M., Han T. (2023). Grazing practices affect soil microbial networks but not diversity and composition in alpine meadows of northeastern Qinghai-Tibetan Plateau. Environ. Res..

[43] Xiang M., Liang Z., Zhang Y., Wu J., Ma T., Duo L., Zhang X., Fu G. (2025). Grazing intensity modifies soil microbial diversity and their co-occurrence networks in an alpine steppe, central Tibet. Microorganisms.

[44] Fang B.Z., Salam N., Han M.X., Jiao J.-Y., Cheng J., Wei D.Q., Xiao M., Li W.J. (2017). Insights on the effects of heat pretreatment, pH, and calcium salts on isolation of rare Actinobacteria from karstic caves. Front. Microbiol..

[45] Shoemaker L.G., Sullivan L.L., Donohue I., Cabral J.S., Williams R.J., Mayfield M.M., Chase J.M., Chu C., Harpole W.S., Huth A. (2020). Integrating the underlying structure of stochasticity into community ecology. Ecology.

[46] Zhang B., Xue K., Zhou S., Wang K., Liu W., Xu C., Cui L., Li L., Ran Q., Wang Z. (2022). Environmental selection overturns the decay relationship of soil prokaryotic community over geographic distance across grassland biotas. eLife.

[47] Liu W., Graham E.B., Dong Y., Zhong L., Zhang J., Qiu C., Chen R., Lin X., Feng Y. (2021). Balanced stochastic versus deterministic assembly processes benefit diverse yet uneven ecosystem functions in representative agroecosystems. Environ. Microbiol..

[48] Li Y.Z., Bao X.L., Zhu X.F., Deng F.B., Yang Y.L., Zhao Y., Xie H.T., Tang S.X., Ge C.J., Liang C. (2024). Parent material influences soil properties to shape bacterial community assembly processes, diversity, and enzyme-related functions. Sci. Total Environ..

[49] Albright M.B.N., Martiny J.B.H. (2018). Dispersal alters bacterial diversity and composition in a natural community. ISME J..

[50] Stegen J.C., Lin X., Fredrickson J.K., Konopka A.E. (2015). Estimating and mapping ecological processes influencing microbial community assembly. Front. Microbiol..

[51] Ofiţeru I.D., Lunn M., Curtis T.P., Wells G.F., Criddle C.S., Francis C.A., Sloan W.T. (2010). Combined niche and neutral effects in a microbial wastewater treatment community. Proc. Natl. Acad. Sci. USA.

[52] Kaisermann A., Maron P., Beaumelle L., Lata J. (2015). Fungal communities are more sensitive indicators to non-extreme soil moisture variations than bacterial communities. Appl. Soil Ecol..

[53] Zhou S., Lie Z., Liu X., Zhu Y., Peñuelas J., Neilson R., Su X., Liu Z., Chu G., Meng Z. (2023). Distinct patterns of soil bacterial and fungal community assemblages in subtropical forest ecosystems under warming. Glob. Change Biol..

[54] Graham E.B., Crump A.R., Resch C.T., Fansler S., Arntzen E., Kennedy D.W., Fredrickson J.K., Stegen J.C. (2016). Coupling spatiotemporal community assembly processes to changes in microbial metabolism. Front. Microbiol..

[55] Yang L., Ning D., Yang Y., He N., Li X., Cornell C.R., Bates C.T., Filimonenko E., Kuzyakov Y., Zhou J. (2022). Precipitation balances deterministic and stochastic processes of bacterial community assembly in grassland soils. Soil Biol. Biochem..

[56] Evans S.E., Bell-Dereske L.P., Dougherty K.M., Kittredge H.A. (2020). Dispersal alters soil microbial community response to drought. Environ. Microbiol..

[57] Maestre F.T., Delgado-Baquerizo M., Jeffries T.C., Eldridge D.J., Ochoa V., Gozalo B., Quero J.L., García-Gómez M., Gallardo A., Ulrich W. (2015). Increasing aridity reduces soil microbial diversity and abundance in global drylands. Proc. Natl. Acad. Sci. USA.

[58] Wang Y., Li C., Tu B., Kou Y., Li X. (2021). Species pool and local ecological assembly processes shape the β-diversity of diazotrophs in grassland soils. Soil Biol. Biochem..

[59] Luan L., Liang C., Chen L., Wang H., Xu Q., Jiang Y., Sun B. (2020). Coupling bacterial community assembly to microbial metabolism across soil profiles. mSystems.

[60] Delgado-Baquerizo M., Fry E.L., Eldridge D.J., de Vries F.T., Manning P., Hamonts K., Kattge J., Boenisch G., Singh B.K., Bardgett R.D. (2018). Plant attributes explain the distribution of soil microbial communities in two contrasting regions of the globe. New Phytol..

