# Supplementary material for: Divergent Association Pathways of Soil Organic Carbon Variation Under Grazing Exclusion Across Three Grassland Sites: Relationships with Microbial Network Structure and Community Assembly
Source: Microorganisms. 2026 Sep 11;14(9):2023. doi: 10.3390/microorganisms14092023 (PMC13609439; doi:10.3390/microorganisms14092023)
Supplement: Supplementary file 1 [file microorganisms-14-02023-s001.zip › microorganisms-4435625-supplementary.pdf]

Supporting information

1.1 Effects of enclosure on plant, soil, and microbial characteristics across different grassland types

Table S1. Effects of enclosure on plant, soil, and microbial characteristics of different grassland types

Variable	TD		TS		MM	
	FG	GE	FG	GE	FG	GE
Richness index	2.11±0.21a	1.94±0.15a	**7.40±0.42b**	**10.93±0.45a**	**8.47±0.35b**	**11.2±0.80a**
Shannon-Wiener index	0.56±0.09a	0.50±0.08a	**1.66±0.08b**	**2.17±0.05a**	**1.89±0.05b**	**2.22±0.09a**
Above-ground biomass ($g \cdot m^{-2}$)	16.91±3.20a	12.31±2.28a	**45.36±4.38b**	**109.07±7.49a**	**101.25±5.56b**	**179.01±17.21a**
Below-ground biomass ($g \cdot m^{-2}$)	**405±6.52a**	**303±19.21b**	**2048±45.13b**	**2497±54.81a**	**1173±63.32b**	**2153±51.61a**
pH	9.23±0.08a	9.38±0.08a	**7.33±0.07a**	**6.95±0.05b**	7.13±0.07a	7.25±0.03a
SWC (%)	**3±0.01b**	**6.20±0.20a**	**22.20±0.37b**	**30.60±1.44a**	**17.20±0.49b**	**25.2±0.49a**
TN ($g \cdot kg^{-1}$)	**0.18±0.01b**	**0.26±0.01a**	**5.38±0.05b**	**5.83±0.10a**	**3.38±0.16b**	**4.23±0.07a**
TP ($g \cdot kg^{-1}$)	**0.28±0.02b**	**0.38±0.01a**	1.08±0.04a	1.11±0.05a	**0.81±0.06b**	**1.14±0.01a**
MBC ($mg \cdot kg^{-1}$)	**42.24±2.92b**	**59±1.11a**	**638.36±14.17b**	**779.66±12.16a**	**394.28±10.20b**	**490.26±4.25a**
MBN ($mg \cdot kg^{-1}$)	**5.89±0.53b**	**7.72±0.42a**	**52.54±0.42b**	**61.19±1.17a**	**16.37±0.50b**	**47.67±0.85a**
MBP ($mg \cdot kg^{-1}$)	**1.04±0.07b**	**1.36±0.02a**	**13.23±0.94b**	**17±0.91a**	**9.48±1.01b**	**12.88±0.14a**

Note: Different lowercase letters indicate significant differences between freely grazed (FG) and grazing exclusion (GE) plots within the same grassland type ($P < 0.05$). Bold data indicated significant effects ($P<0.05$), TD, TS, MM, GE and FG respectively represent temperate desert, temperate steppe, mountain meadow, grazing exclusion plots and freely grazing plots.

1.2 Effects of enclosure on microbial community structure of different grassland types

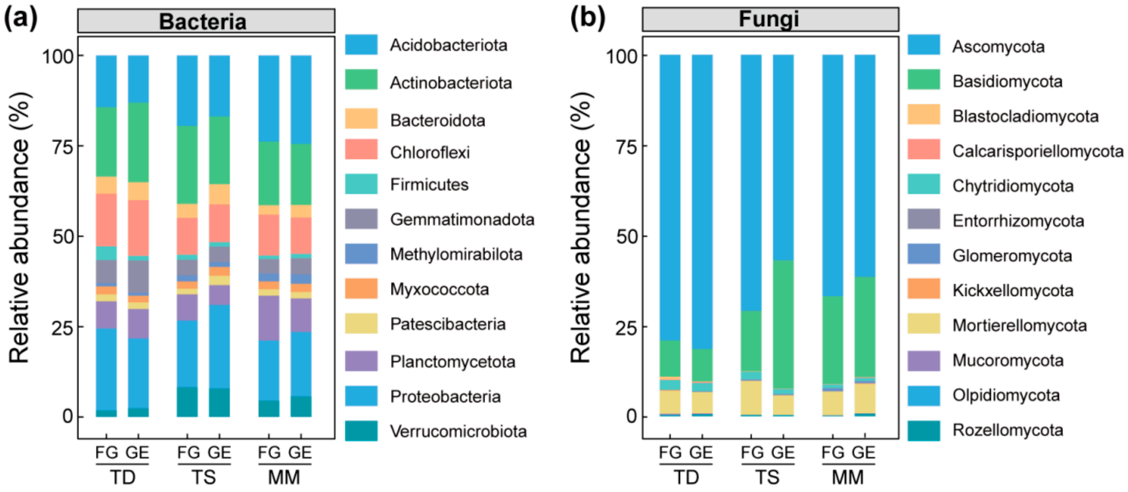

Figure S1. Effects of enclosure on microbial community structure at phylum level of different grassland types. Note: (a) Relative abundance of bacterial phyla under grazing exclusion (GE) and free grazing (FG). (b) Relative abundance of fungal phyla under GE and FG. TD, TS, MM, GE and FG respectively represent temperate desert, temperate steppe, mountain meadow, grazing exclusion plots and freely grazing plots.

1.3 Effects of enclosure on microbial community diversity of different grassland types

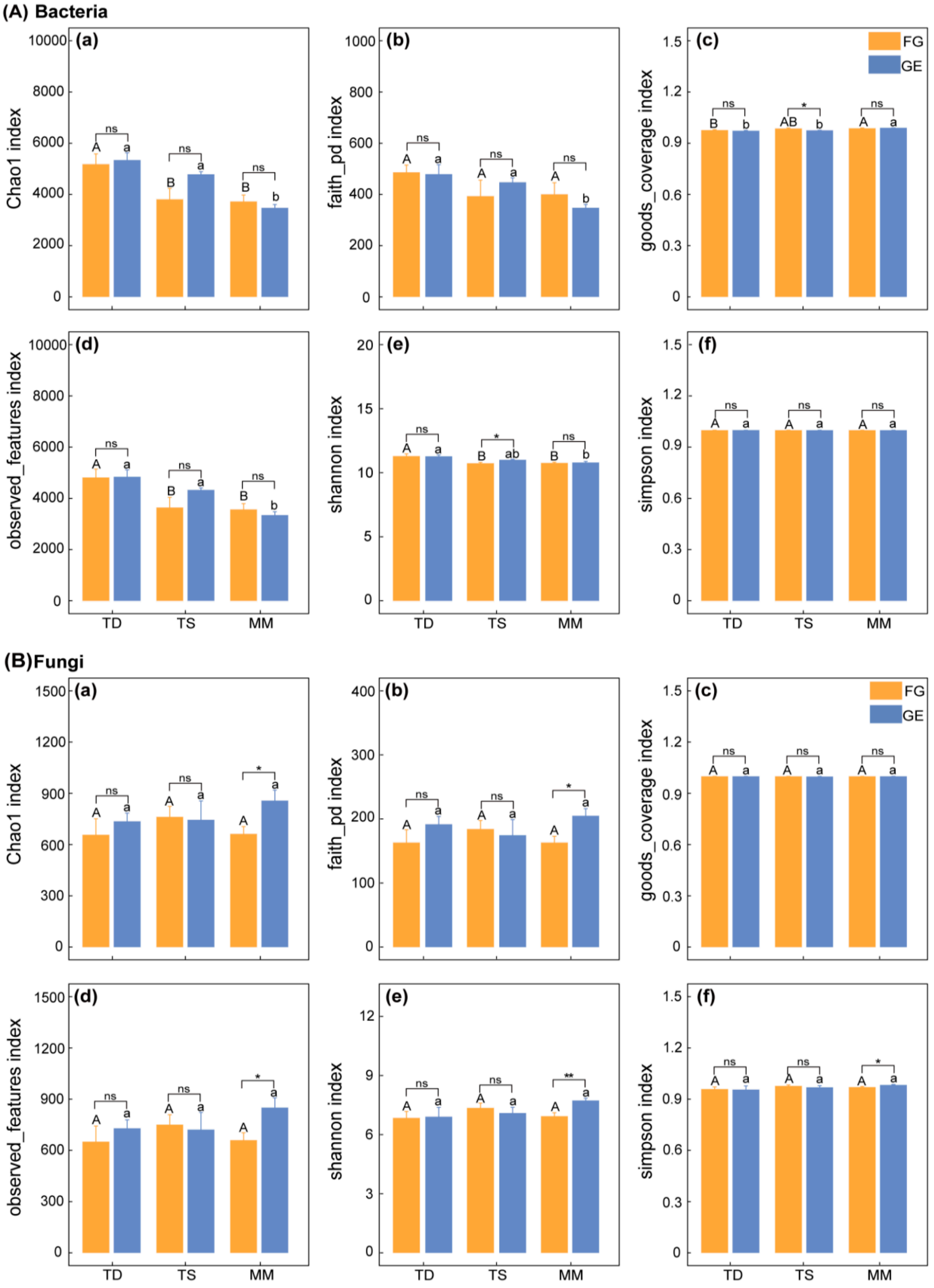

Figure S2. Effects of enclosure on microbial community diversity of different grassland types Note: (A) Bacterial diversity indices: (a) Chao1 index; (b) faith_pd index; (c) goods coverage index; (d) observed_features index; (e) Shannon index; (f) Simpson index. (B) Fungal diversity indices: (a) Chao1 index; (b) faith_pd index; (c) goods coverage index; (d) observed_features index; (e) Shannon index; (f) Simpson index. All data points and error bars in histograms represent mean ± standard error. "*", "**", and "***" indicate significant differences at $P < 0.05$, $P < 0.01$, and $P < 0.001$, respectively, between grazing exclusion and freely grazed treatments within the same grassland type, as determined by appropriate two-sided statistical tests based on normality and variance assumptions. "ns" indicates no significant difference ($P > 0.05$). Capital letters indicate significant differences among grassland types within the freely grazed treatment ($P < 0.05$), as determined by one-way ANOVA followed by Tukey's HSD test. Lowercase letters

indicate significant differences among grassland types within the grazing exclusion treatment ($P < 0.05$), as determined by one-way ANOVA followed by Tukey's HSD test; shared letters indicate no significant differences ($P > 0.05$). TD, temperate desert; TS, temperate steppe; MM, mountain meadow; GE, grazing exclusion plots; FG, freely grazed plots.
